# Synthesis and pre-clinical evaluation of a new class of high-affinity ^18^F-labeled PSMA ligands for detection of prostate cancer by PET imaging

**DOI:** 10.1007/s00259-016-3556-5

**Published:** 2016-11-15

**Authors:** James Kelly, Alejandro Amor-Coarasa, Anastasia Nikolopoulou, Dohyun Kim, Clarence Williams, Shashikanth Ponnala, John W. Babich

**Affiliations:** 1 Division of Radiopharmaceutical Sciences and Molecular Imaging Innovations Institute, Department of Radiology, Weill Cornell Medicine, Belfer Research Building, Room 1600, 413 East 69th Street, New York, NY 10021 USA; 2 Citigroup Biomedical Imaging Center, Weill Cornell Medicine, New York, NY 10021 USA; 3 Meyer Cancer Center, Weill Cornell Medicine, New York, NY 10021 USA

**Keywords:** Prostate cancer, Triazolylphenyl ureas, Fluorine-18, PSMA

## Abstract

**Purpose:**

Current clinical imaging of PSMA-positive prostate cancer by positron emission tomography (PET) mainly features ^68^Ga-labeled tracers, notably [^68^Ga]Ga-PSMA-HBED-CC. The longer half-life of fluorine-18 offers significant advantages over Ga-68, clinically and logistically. We aimed to develop high-affinity PSMA inhibitors labeled with fluorine-18 as alternative tracers for prostate cancer.

**Methods:**

Six triazolylphenyl ureas and their alkyne precursors were synthesized from the Glu-urea-Lys PSMA binding moiety. PSMA affinity was determined in a competitive binding assay using LNCaP cells. The [^18^F]triazoles were isolated following a Cu(I)-catalyzed click reaction between the alkynes and [^18^F]fluoroethylazide. The ^18^F-labeled compounds were evaluated in nude mice bearing LNCaP tumors and compared to [^68^Ga]Ga-PSMA-HBED-CC and [^18^F]DCFPyL. Biodistribution studies of the two tracers with the highest imaged-derived tumor uptake and highest PSMA affinity were undertaken at 1 h, 2 h and 4 h post-injection (p.i.), and co-administration of PMPA was used to determine whether uptake was PSMA-specific.

**Results:**

F-18-labeled triazolylphenyl ureas were prepared with a decay-corrected RCY of 20–40 %, >98 % radiochemical and chemical purity, and specific activity of up to 391 GBq/μmol. PSMA binding (IC_50_) ranged from 3–36 nM. The position of the triazole influenced tumor uptake (3 > 4 > 2), and direct conjugation of the triazole with the phenylurea moiety was preferred to insertion of a spacer group. Image-derived tumor uptake ranged from 6–14 %ID/g at 2 h p.i., the time of maximum tumor uptake; uptake of [^68^Ga]Ga-PSMA-HBED-CC and [^18^F]DCFPyL was 5–6 %ID/g at 1–3 h p.i., the time of maximum tumor uptake. Biodistribution studies of the two most promising compounds gave maximum tumor uptakes of 10.9 ± 1.0 % and 14.3 ± 2.5 %ID/g, respectively, as compared to 6.27 ± 1.44 %ID/g for [^68^Ga]Ga-PSMA-HBED-CC.

**Conclusions:**

Six [^18^F]triazolylphenyl ureas were prepared in good radiochemical yield. Compounds showed PSMA-specific uptake in LNCaP tumors as high as 14 % ID/g, more than a 2-fold increase over [^68^Ga]Ga-PSMA-HBED-CC. The facile and high-yielding radiosynthesis of these ^18^F-labeled triazoles as well as their promising in vitro and in vivo characteristics make them worthy of clinical development for PET imaging of prostate cancer.

**Electronic supplementary material:**

The online version of this article (doi:10.1007/s00259-016-3556-5) contains supplementary material, which is available to authorized users.

## Introduction

Prostate cancer is the second most prevalent cancer among men, with more than 1.1 million diagnoses in 2012 [1] and more than 292,000 deaths due to prostate cancer reported worldwide in 2013 [2]. The disease burden continues to grow—157,000 deaths were reported in 1990 [2]—and it is estimated that more than 180,000 men will be newly diagnosed with prostate cancer in the United States in 2016, and that more than 26,000 deaths due to the disease will be registered [3]. When detected early and the disease is confined to the prostate gland and regional lymph nodes, the 5-year survival rate is nearly 100 %, but the survival rate drops below 30 % when the disease is metastatic [4]. Early diagnosis can significantly improve patient prognosis, while sensitive and specific localization of the disease is an important feature in the diagnosis and staging of the disease. Accurate staging is critical for appropriate patient management [5].

Prostate-specific membrane antigen (PSMA; also known as glutamate carboxypeptidase II) is significantly overexpressed in prostate cancer primary tumors and many metastatic lesions, while expression in healthy prostate and other tissue is limited [6]. Several other characteristics combine to make PSMA an ideal target for molecular diagnostics and therapeutics for prostate cancer: (1) it is overexpressed at all stages of the disease; (2) expression typically correlates with tumor grade, disease aggressiveness, metastasis and biochemical recurrence; (3) it is a transmembrane protein with an extracellular ligand-binding domain; and (4) the bound ligand-protein complex is internalized via receptor-mediated clathrin-dependent endocytosis [7, 8]. The potential utility of PSMA as a target for diagnostic imaging and therapy was demonstrated with the radiolabeled monoclonal antibody J591 [9], using In-111 or Zr-89 for imaging and Y-90 or Lu-177 for therapy, however the pharmacokinetics of the antibody make it unsuitable for diagnostic imaging with short-lived radionuclides [10].

A number of low molecular weight, urea-based small molecules have been described as single photon emission computed tomography (SPECT) or positron emission tomography (PET) imaging agents for prostate cancer, and several of them are undergoing clinical investigation in humans (structures can be found in Fig. S1 of the Supplementary Materials). Currently, seven such molecules are in Phase I/II clinical trials in the United States and/or Europe: (i) radioiodinated MIP-1095 (I-123 for SPECT/CT and I-124 for PET/CT) and MIP-1072 (I-123 for SPECT/CT), developed by Molecular Insight Pharmaceuticals, Inc., (ii) ^99m^Tc-MIP-1404 and ^99m^Tc-MIP-1405, two further SPECT imaging agents emerging from the Molecular Insight Pharmaceuticals platform, (iii) [^68^Ga]Ga-PSMA-HBED-CC (also known as [^68^Ga]PSMA-11 and [^68^Ga]DKFZ-PSMA-11) for PET/CT, and (iv) [^18^F]DCFBC and its next-generation derivative [^18^F]DCFPyL for PET/CT [11, 12]. Newly introduced compounds to undergo first-in-human evaluation include ^68^Ga-DKFZ-617, developed to be a theranostic ligand and evaluated in a therapeutic context as ^177^Lu-DKFZ-617 [13], and the structurally related ^68^Ga-CHX-A”-DTPA [14].

The greater sensitivity and higher spatial resolution of PET relative to SPECT has made this technique the preferred imaging platform in a number of clinical environments [15, 16]. Among the positron-emitting isotopes currently incorporated into PSMA-targeting ligands, fluorine-18 and gallium-68 are preferred to iodine-124 because of their higher efficiency of positron emission (97 % and 89 % vs. 23 %, respectively) and shorter half-lives. Furthermore, iodine-124 scans require complex reconstruction algorithms to minimize the signal-to-noise ratio, which, in combination with the long half-life of iodine-124 (t_1/2_ = 4.18 d) and the undesired emission of beta particles, is often a poor match for the pharmacokinetics of small molecules [17]. In addition, gallium-68 is currently produced from a ^68^Ge/^68^Ga generator, enabling its use in single-batch syntheses in radiopharmacies independent of access to a cyclotron, and chelation of gallium-68 is both clean and rapid under conditions that are compatible with most small molecules and peptides. These considerations have contributed to the emergence of [^68^Ga]Ga-PSMA-HBED-CC as the most widely used radiotracer currently in clinical development [18].

Fluorine-18 presents a number of practical advantages compared to gallium-68, including: (i) a longer half-life [t_1/2_(^18^F) = 109.8 min vs. t_1/2_(^68^Ga) = 67.7 min], which permits multiple step radiosyntheses and allows a longer time for background signals to clear before imaging is performed, (ii) large-scale cyclotron production that allows multiple patient doses to be produced from a single synthesis, and (iii) chemical characteristics, such as a similar atomic radius to hydrogen, that allow diverse types of ligands to be prepared. On this basis, the development of PSMA-targeting ligands labeled with fluorine-18 has emerged recently as a goal of great interest. [^18^F]DCFBC is based on a Glu-urea-Cys pharmacophore modified with a 4-[^18^F]fluorobenzyl group, and was reported to show good uptake in PSMA^+^ xenograft tumors [19]. In humans, however, it has shown limited clearance from soft tissue, resulting in a decreased tumor-to-background ratio and poor visualization of small lesions [20, 21]. To address the slow clearance, the second-generation [^18^F]fluoropyridine-modified Glu-urea-Lys analogue [^18^F]DCFPyL was developed [22]. Despite more promising pharmacokinetics, fast tumor washout is evident as early as 1 h post-injection (p.i.) and accumulation of radioactivity in evacuated bladders is considerable [22]. In addition, [^18^F]DCFPyL suffers from poor radiochemical yields (in the range 5–12 % decay-corrected [23, 24], although recently an improved synthesis by direct fluorination has been reported to increase yield to greater than 20 % [25].

In an effort to address the aforementioned challenges in the development of fluorine-18-PSMA ligands of high specificity and affinity, we describe the synthesis and preliminary structure-activity relationship (SAR) studies of two new classes of [^18^F]fluoroethyltriazolylphenyl urea-based PSMA ligands, afforded by click chemistry in high radiochemical yield (20–40 %), excellent radiochemical purity (>99 %) and high specific activity (182–391 GBq/μmol) from starting activities of less than 7.4 GBq (200 mCi). Each of these ligands shows substantial tumor uptake in nude mice bearing LNCaP xenografts using μPET/CT. The two most promising ligands, RPS-040 and RPS-041, show excellent PSMA imaging characteristics based on their high specificity for PSMA, high tumor uptake and prolonged tumor retention, rapid clearance from non-target tissues and resulting high tumor-to-background ratios. They also show superior pharmacokinetics when compared to [^68^Ga]Ga-PSMA-HBED-CC in mice, thereby warranting development as clinical PET imaging agents for prostate cancer.

## Materials and methods

### Synthesis of alkyne precursors

**Fig. 1:**
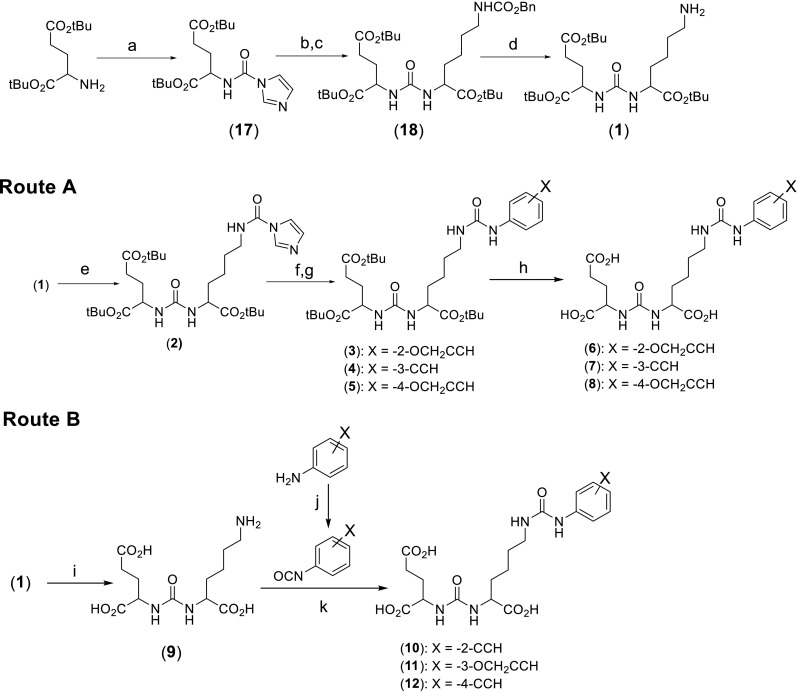
Synthesis of phenylureas derivatized with alkynes as precursors for click chemistry

The alkyne precursors were synthesized by one of two routes, described in Fig. 1. In route A, the phenylurea moiety was synthesized by coupling an alkyne-substituted aniline with the protected Glu-urea-Lys group [26] via 2-carbonyldiimidazole, with acid-catalyzed deprotection giving the alkyne precursor. In route B, the aniline was converted to an isocyanate via triphosgene and then treated with the deprotected Glu-urea-Lys group. This route avoided acid-catalyzed deprotection of acid-sensitive compounds. Full experimental details can be found in the Supplementary Materials.

### Synthesis of fluorinated triazoles

**Fig. 2:**
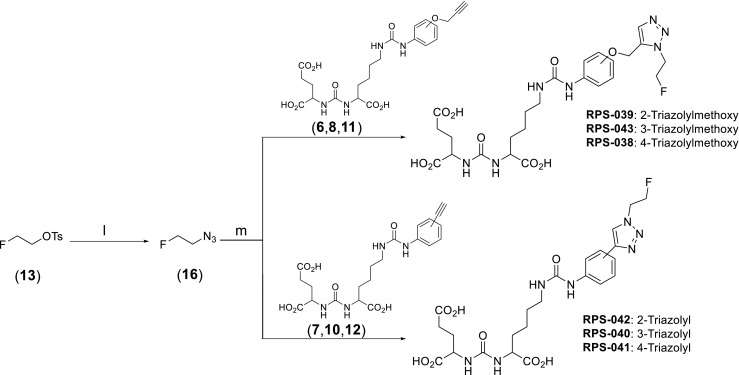
Synthesis of two classes of phenylureas derivatized with triazoles. Series one contains RPS-039, RPS-043 and RPS-038. Series two contains RPS-042, RPS-040 and RPS-041

The reference triazoles were synthesized from 2-fluoroethylazide [27] and the corresponding alkyne as described in Fig. 2. Full experimental details can be found in the Supplementary Materials.

### Synthesis of 2-azidoethyltosylate

**Fig. 3:**
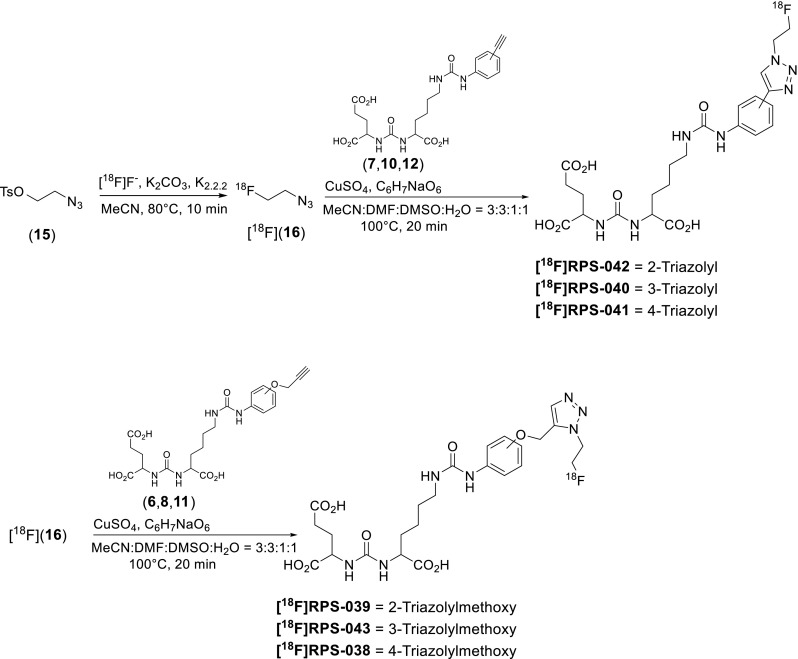
Two-step radiosynthesis of six phenylureas by click chemistry

The 2-azidoethyltosylate (**15**) synthon for ^18^F-fluorination (Fig. 3) was synthesized in two steps from 2-bromoethanol [28]. A nucleophilic substitution reaction involving sodium azide generated 2-azidoethanol, with tosylation by *p*-toluenesulfonyl chloride providing the precursor for radiolabeling. Full experimental details can be found in the Supplementary Materials.

### Radiosynthesis

#### General methods

All solvents and reagents were purchased from Sigma-Aldrich and were of reagent grade quality unless otherwise indicated. All reactions were carried out in oven-dried glassware. Fluorine-18 was obtained by irradiation of H_2_^18^O (Rotem Industries) via the ^18^O(p,n)^18^F transformation using a TR19 cyclotron (Advanced Cyclotron Systems, Inc.). End-of-bombardment activity was typically 5.55-9.25 GBq (150–250 mCi). Analytical and semi-preparative high-performance liquid chromatography (HPLC) were performed on a dual pump Varian high-performance liquid chromatography (Agilent Technologies) fitted with a dual ultraviolet-visible light (UV–vis) detector and an NaI(Tl) detector (Bioscan). Solvent A was 0.01 % trifluoroacetic acid (TFA) in H_2_O and solvent B was 0.01 % TFA in 90:10 v/v MeCN:H_2_O. Semi-prep HPLC was performed on a Bondapak C18 7.8 x 300-mm, 125-Å column (Waters) while analytical HPLC was performed on a Symmetry C18 4.6 x 50-mm, 100-Å column (Waters). The UV absorption spectrum was monitored at 220 nm and 280 nm. Semi-prep HPLC was performed using an isocratic solvent mixture of 15 % B at a flow rate of 4 mL/min. Analytical HPLC was generally performed at a flow rate of 2 ml/min using the following gradient; 0%B 0–1 min., 0–100%B 1–8 mins., 100–0%B 8–10 mins.

All radiochemical yields were corrected to the [^18^F]fluoride activity measured at start-of-synthesis. The reaction conditions reported represent the highest yields obtained using manual radiosyntheses. The radiosynthesis of [^68^Ga]Ga-PSMA-HBED-CC [29] and [^18^F]DCFPyL [22] were performed according to previously reported methods and are described in the supporting information.

#### Radiosynthesis of the ^18^F-RPS series

No-carrier-added [^18^F]fluoride was trapped on a pre-activated Sep-Pak QMA cartridge (Waters) and eluted with 1 mL of an 80 % v/v MeCN/H_2_O solution containing 2.7 mg K_2_CO_3_ and 4 mg Kryptofix-222. The solution was dried azeotropically with MeCN (2 x 0.5 mL) at 100 °C in 10 min. To the dried F-18 was added a solution of 2-azidoethyltosylate (**15**; 6 mg) in MeCN (300 μL). The resulting solution was stirred at 80 °C for 10 min to yield 2-[^18^F]fluoroethylazide. The 2-[^18^F]fluoroethylazide was purified by distillation by heating the vial at 130 °C and trapping the 2-[^18^F]fluoroethylazide in a vial containing 100 μL dimethylformamide (DMF) cooled to 0 °C.

A pre-mixed solution of 0.5 M CuSO_4_ (50 μL) and 1.5 M sodium ascorbate (50 μL) in DMF (100 μL) was added to the vial containing the 2-[^18^F]fluoroethylazide solution followed by 1 mg of alkyne precursor (**6**–**8**; **10**–**12**) in dimethyl sulfoxide (DMSO; 100–150 μL). The reaction was stirred at 100 °C for 20 min. It was then cooled to room temperature, diluted with 2 mL H_2_O and filtered through a 0.45-μm nylon syringe filter (Cole-Parmer). The filter was washed with 1 mL H_2_O, which was added to the filtrate. The filtrate was purified by semi-prep reverse-phase HPLC (4 mL/min; 0–100 % B; 30 min), and the peak corresponding to ^18^F-labeled triazole was collected, diluted with H_2_O and passed through a pre-activated Oasis™ solid-phase extraction cartridge (Waters). The retained activity was eluted with ethanol (EtOH) and diluted with 0.9 % NaCl solution until the concentration of ethanol was less than 5 % v/v and the radioactivity concentration was a minimum of 74 MBq/mL. The synthesis, purification and final formulation were achieved in 105 min from start-of-synthesis. An optimized isocratic HPLC purification method (4 mL/min; 15 % B; 30 min) was used to isolate [^18^F]RPS-040 and [^18^F]RPS-041 in 20–40 % decay-corrected radiochemical yield, greater than 99 % radiochemical purity and a specific activity of up to 391 GBq/μmol.

## Cell culture

The human prostate cancer cell line, LNCaP, was obtained from the American Type Culture Collection. Cell culture supplies were from Invitrogen unless otherwise noted. LNCaP cells were maintained in RPMI-1640 medium supplemented with 10 % fetal bovine serum (Hyclone), 4 mM L-glutamine, 1 mM sodium pyruvate, 10 mM N-2-hydroxyethylpiperazine-N-2-ethanesulfonic acid (HEPES), 2.5 mg/mL D-glucose, and 50 μg/mL gentamicin in a humidified incubator at 37 °C/5 % CO_2_. Cells were removed from flasks for passage or for transfer to 12-well assay plates by incubating them with 0.25 % trypsin/ethylenediaminetetraacetic acid (EDTA).

### In vitro determination of IC_50_

The 50 % inhibition concentrations (IC_50_ ) values of the non-radioactive fluorine-containing ligands were determined by screening in a multi-concentration competitive binding assay against ^99m^Tc- ((7S,12S,16S)-1-(1-(carboxymethyl)-1H-imidazol-2-yl)-2-((1-(carboxymethyl)-1H-imidazol-2-yl)methyl)-9,14-dioxo-2,8,13,15-tetraazaoctadecane-7,12,16,18-tetracarboxylic acid technetium tricarbonyl complex; ^99m^Tc-MIP-1427) for binding to PSMA on LNCaP cells, according to methods previously described [30]. The LNCaP cells were plated 48 hours prior to the experiment to achieve a density of approximately 5 x 10^5^ cells/well (in triplicate) in RPMI-1640 medium supplemented with 0.25 % bovine serum albumin prior to performing the assay. LNCaP cells were incubated for 1 hour with 1 nM ^99m^Tc-MIP-1427 in serum-free RPMI-1640 medium in the presence of 1–10,000 nM test compounds. Radioactive incubation media was then removed by pipette and the cells were washed twice using 1 mL of ice-cold HEPES buffer. Cells were harvested from the plates and transferred to tubes for radioactive counting using a Packard Cobra II gamma counter. IC_50_ values were determined by non-linear regression using GraphPad Prism software.

### Inoculation of mice with xenografts

All animal studies were approved by the Institutional Animal Care and Use Committee of Weill Cornell Medicine and were undertaken in accordance with the guidelines set forth by the USPHS Policy on Humane Care and Use of Laboratory Animals. Animals were housed under standard conditions in approved facilities with 12-h light/dark cycles. Food and water was provided ad libitum throughout the course of the studies. Male inbred athymic nu/nu mice were purchased from The Jackson Laboratory. For inoculation in mice, LNCaP cells were suspended at 4 x 10^7^ cells/mL in a 1:1 mixture of PBS:Matrigel (BD Biosciences). Each mouse was injected in the left flank with 0.25 mL of the cell suspension. The mice were imaged when the tumors reached approximately 200–400 mm^3^, while biodistributions were conducted when tumors were in the range 100–400 mm^3^.

### Imaging

LNCaP xenograft tumor-bearing mice (two per compound) were injected intravenously via the tail vein as a bolus injection of 7.03–7.77 MBq (190–210 μCi) of the tracer ([^18^F]RPS series), 5.5–6.5 MBq (150–175 μCi) [^18^F]DCFPyL or 5.5 MBq (150 μCi) [^68^Ga]Ga-PSMA-HBED-CC. Specific activity was greater than 190 GBq/μmol. The mice were imaged by μPET/CT (Inveon™; Siemens Medical Solutions, Inc.) at 1 h, 2 h, 4 h and 6 h p.i. ([^18^F]fluorinated compounds) or 1 h and 3 h p.i. ([^68^Ga]Ga-PSMA-HBED-CC). Total acquisition time was thirty minutes, and a CT scan was obtained either immediately before or immediately after the acquisition for both anatomical co-registration and attenuation correction. The data were reconstructed using the commercial Inveon™ software supplied by the vendor. Tumor uptake was estimated by drawing a region of interest (ROI).

### Biodistribution

LNCaP xenograft tumor-bearing mice (n = 5 per time point) were injected via the tail vein with a bolus injection of 370 kBq (10 μCi) of either [^18^F]RPS-040 or [^18^F]RPS-041. The specific activity of the compounds was 341 GBq/μmol and 391 GBq/μmol, respectively. The mice were euthanized by asphyxiation under isofluorane at 1 h, 2 h and 4 h p.i. An additional set of mice (n = 5) was co-administered [^18^F]RPS-040 (370 kBq; 10 μCi) and 2-PMPA (approx. 250 μg; 10 mg/kg) and sacrificed at 1 h p.i. to determine the uptake specificity. A full biodistribution study was conducted on all mice, and tissues were excised, weighed and counted in an automated γ-counter. Tissue time-activity values were expressed as percentage injected dose per gram of tissue (%ID/g). Statistical comparisons were performed using the standard Student’s *t* test for a 95 % confidence interval.

## Results

### Synthesis and radiosynthesis

The synthetic schema of the six ^18^F-fluorinated PSMA inhibitors [^18/19^F]RPS-038 to [^18/19^F]RPS-043 is given in Figs. 1–3. Full experimental details, including a description of unexpected acid-catalyzed degradation of certain alkyne precursors that necessitated an alternative synthetic route, are available in the Supplementary Information.

The two classes of alkyne precursors were synthesized via different routes due to the instability of some of the alkyne precursors towards the acidic deprotection of the *tert*-butyl esters. The 2- and 4-((propenyloxy)phenyl)urea (**6**, **8**) and 3-((ethynyl)phenyl)urea (**7**) precursors were largely stable to acidic deprotection and so were synthesized in three steps from the protected Glu-urea-Lys (**1**) intermediate. Some degradation of the 3-((ethynyl)phenyl)urea was observed during deprotection, but the major product was the desired alkyne. However, the 2- and 4-((ethynyl)phenyl)ureas (**10**, **12**) and the 3-((propenyloxy)phenyl)urea (**11**) required conversion to their corresponding isocyanates with triphosgene, and the crude reaction products were then treated with the free acid form of the Glu-urea-Lys (**9**) pharmacophore (Fig. 1). The yields of the 3-substituted phenylureas (6.6–20.0 % from (**1**)) were relatively poor using either synthetic route, while the 2-substituted phenylureas (27.3–28.2 %) and 4-substituted phenylureas (26.5–33.4 %) were synthesized in greater yields from the same starting point.

The cold ^19^F containing ligands RPS-038–RPS-043 were synthesized by a Cu(I)-catalyzed click reaction with 2-fluoroethylazide (**16**), prepared in situ from 2-fluoroethyltosylate (**13**) and sodium azide (Fig. 2). Following semi-prep HPLC purification, the triazoles RPS-038, RPS-040, RPS-042 and RPS-043 were isolated in 60–82 % yield. The yields of RPS-041 (50 %) and RPS-039 (34 %) were somewhat lower than expected, likely due to potential contamination of the alkyne starting material with inseparable impurities.

The radiosynthetic schema describing the preparation of the ^18^F-containing triazoles from their alkyne precursors (**6**–**8**, **10**–**12**) is provided in Fig. 3. 2-Azidoethyltosylate (**15**) was synthesized in two steps from 2-bromoethanol and added as a solution in MeCN to azeotropically dried [^18^F]fluoride-K_2_CO_3_-kryptofix. Incorporation of [^18^F]fluoride was greater than 90 % (n = 12) by radio-HPLC after 10 minutes at 80 °C. Following distillation at 130 °C, 50.1 ± 11.7 % of the 2-[^18^F]fluoroethylazide ([^18^F](**16**)) was isolated in greater than 95 % radiochemical purity. Up to 40 % of the 2-[^18^F]fluoroethylazide remained in the reaction vial, but the addition of small volumes of MeCN to increase recovery was found to have a detrimental effect on the subsequent click reaction.

The click reaction was carried out in mixtures of DMSO/MeCN and yields were highly sensitive to reaction volume and MeCN content. Conversion to the triazole was 50.5 ± 6.7 % (n = 8; as measured by radio-HPLC) when undertaken at 100 °C for 20 min and when the total reaction volume was 600 μL and the MeCN content was 25 %. When MeCN content increased up to 50 %, conversion dropped below 25 %, while an increase in total reaction volume to 1050 μL with 25 % MeCN content dropped conversion to 30–35 %.

High radiochemical purity (>95 %) preparation of the [^18^F]fluorinated triazole was accomplished after solid-phase C18 extraction, which retained approximately 65 % of the labeled triazole and less than 5 % of the unreacted 2-[^18^F]fluoroethylazide. However, the specific activity of this preparation was low due to contamination with the unreacted alkyne. Therefore, purification by semi-prep HPLC, following filtration of the diluted reaction mixture through a 0.45-μm filter to remove solid residues, was used in preference to solid-phase extraction. Recovery of the [^18^F]fluorinated triazole following filtration and HPLC purification was 76.0 ± 6.9 %. Following this procedure, the [^18^F]fluorinated ligands were isolated in 20–40 % radiochemical yield (decay-corrected to start-of-synthesis) and a specific activity of 182–391 GBq/μmol. Optimization and automation of the radiosynthesis and purification are ongoing.

### In vitro evaluation

The affinity for PSMA was determined in a competitive binding assay using LNCaP cells, and the IC_50_s of the six compounds ranged from 3.2–36.5 nM (Table 1). In the same LNCaP-based assay, MIP-1095 was determined to have an IC_50_ of 0.3 nM while DCFPyL, a fluorinated PSMA inhibitor [22] currently undergoing first-in-human trials in the United States [24] and Europe [23] had an IC_50_ of 22.8 nM (Table 1). [^68^Ga]Ga-PSMA-HBED-CC has been reported to have an IC_50_ of approximately 24 nM for PSMA in a competitive binding assay using LNCaP cells [14, 31].

**Table 1:**
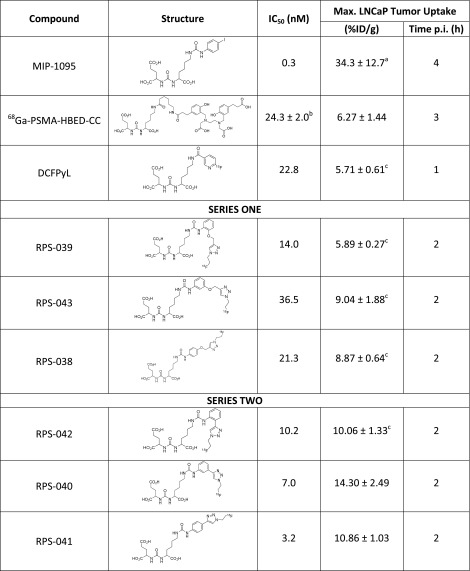
Structure and in vitro affinities for PSMA and HSA of PSMA-binding ligands

a = See Ref [26]

b = See Ref [14]

c = Image derived calculation of tumor uptake

### In vivo evaluation

**Fig. 4:**
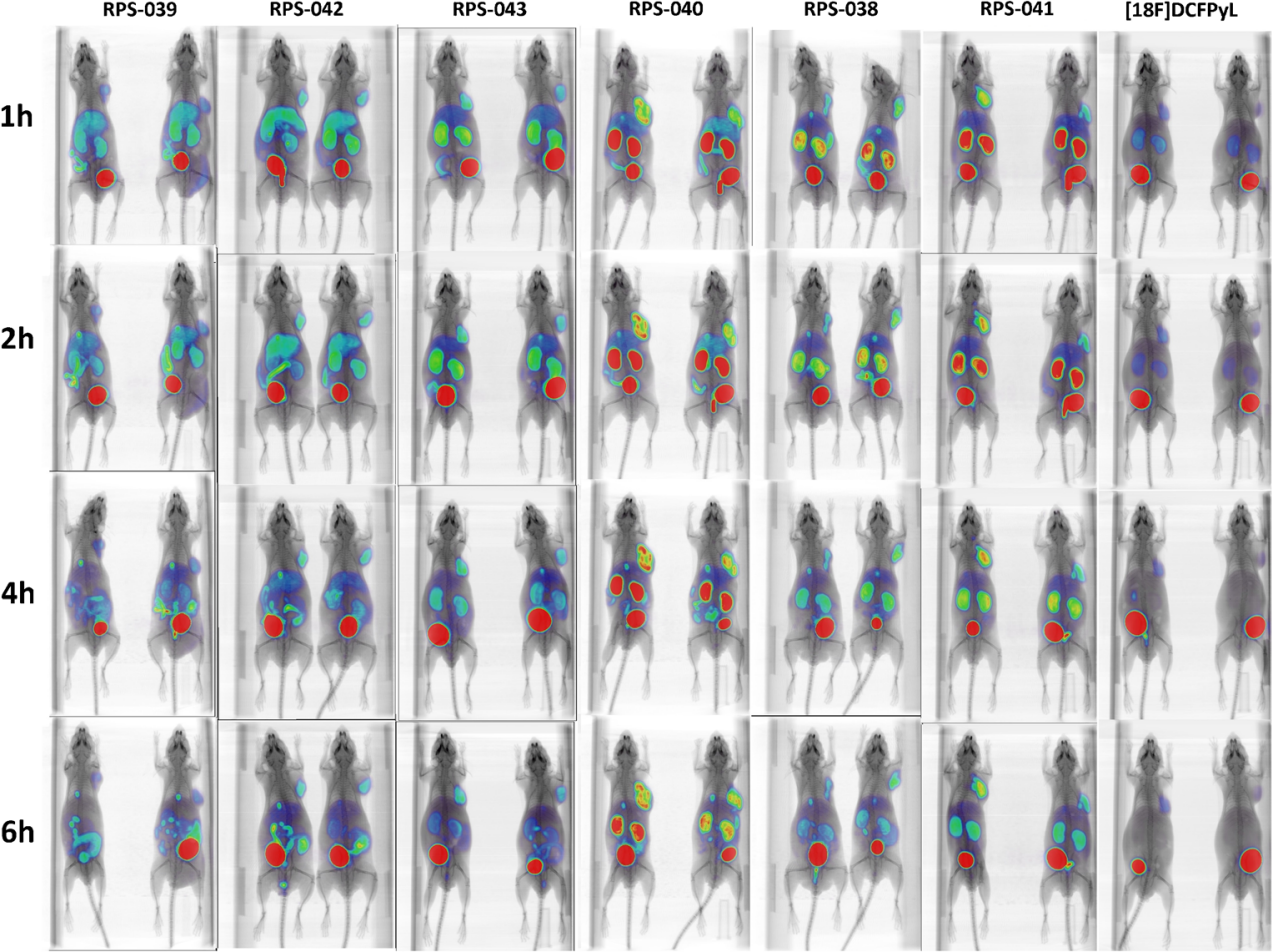
Micro-PET/CT images of a series of [^18^F]fluorinated PSMA ligands in LNCaP xenograft tumor-bearing mice. Mice (n = 2 per time point) were imaged at 1 h, 2 h, 4 h and 6 h p.i. All images are scaled to correct for decay and the highest tumor uptake image (RPS-040; 2 h p.i) is used as the reference for setting the intensity scale

The six compounds were assessed in a mouse model of prostate cancer by μPET/CT imaging at 1 h, 2 h, 4 h and 6 h p.i. Each of the compounds showed good tumor uptake by 1 h, with uptake peaking at 2 h and maintaining a steady value up to 4 h (Fig. 4). No significant washout was observed by 6 h. In contrast, signals in other tissues such as the kidneys and liver began to decrease after 1 h, leading to high-contrast images by 2 h p.i. Excretion was predominantly via the urine as evidenced by the rapid accumulation of activity in the bladder of these mice. Urine was not collected.

The maximum tumor uptake (t = 2 h) for the compounds in series one was estimated from the μPET/CT images to range from 5.89 ± 0.27 %ID/g to 9.04 ± 1.88 %ID/g and with uptake of [^18^F]RPS-043 > [^18^F]RPS-038 > [^18^F]RPS-039 (Table 1). Uptake did not directly correlate with the IC_50_ determined in LNCaP cells, as RPS-039 had the highest affinity for PSMA (14 nM) in series one, but the lowest tumor uptake (5.87 ± 0.27 %ID/g). Compounds in series two showed greater tumor uptake and higher contrast than their series one structural counterparts. Maximum tumor uptake (t = 2 h) was derived from the image and calculated to range from 10.06 ± 1.33 %ID/g to 14.30 ± 0.67 %ID/g and in the order [^18^F]RPS-040 > [^18^F]RPS-041 > [^18^F]RPS-042 (Table 1). It was again the case that the highest affinity compound ([^18^F]RPS-041; IC_50_ = 3.2 nM) did not have the highest tumor uptake.

The major route of clearance appeared to be via the kidneys, with the exception of RPS-039 and RPS-042, which showed clearance via the hepatobiliary pathway in addition to renal clearance. These two compounds share substitution at the 2-position of the phenylurea as a structural feature. While [^18^F]RPS-039 was the most potent series one compound in the in vitro binding assay, both [^18^F]RPS-039 and [^18^F]RPS-042 had the lowest tumor uptake and lowest image contrast of their respective series.

[^18^F]RPS-040 and [^18^F]RPS-041, which had both the highest image-derived tumor uptake (14.30 %ID/g and 12.51 %ID/g, respectively) and greatest tumor/background contrast in the μPET/CT images, were evaluated further by biodistribution studies in LNCaP xenograft tumor-bearing mice.

**Fig. 5:**
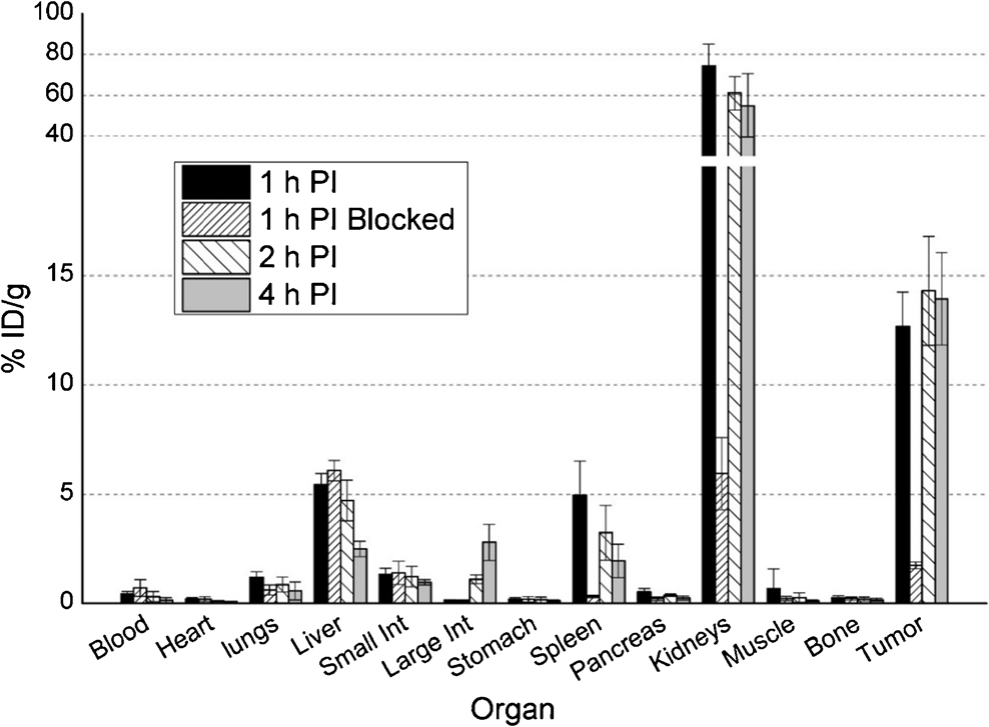
Biodistribution of [^18^F]RPS-040 in LNCaP xenograft tumor-bearing mice. Mice (n = 5 per time point) were sacrificed at 1 h (1 h p.i.), 2 h (2 h p.i.) and 4 h (4 h p.i.) p.i. To determine specificity for PSMA, 2-PMPA was co-administered and the mice were sacrificed at 1 h p.i. (n = 5; 1 h p.i. blocked)

**Fig. 6:**
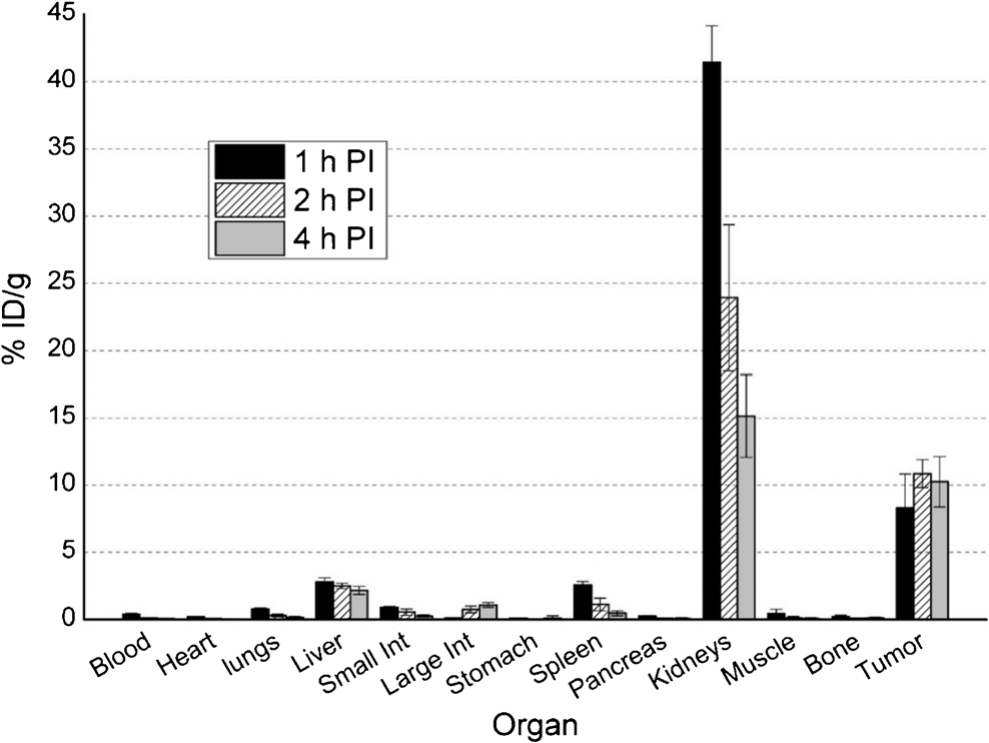
Biodistribution of [^18^F]RPS-041 in LNCaP xenograft tumor-bearing mice. Mice (n = 5 per time point) were sacrificed at 1 h (1 h p.i.), 2 h (2 h p.i.) and 4 h (4 h p.i.) p.i.

**Fig. 7:**
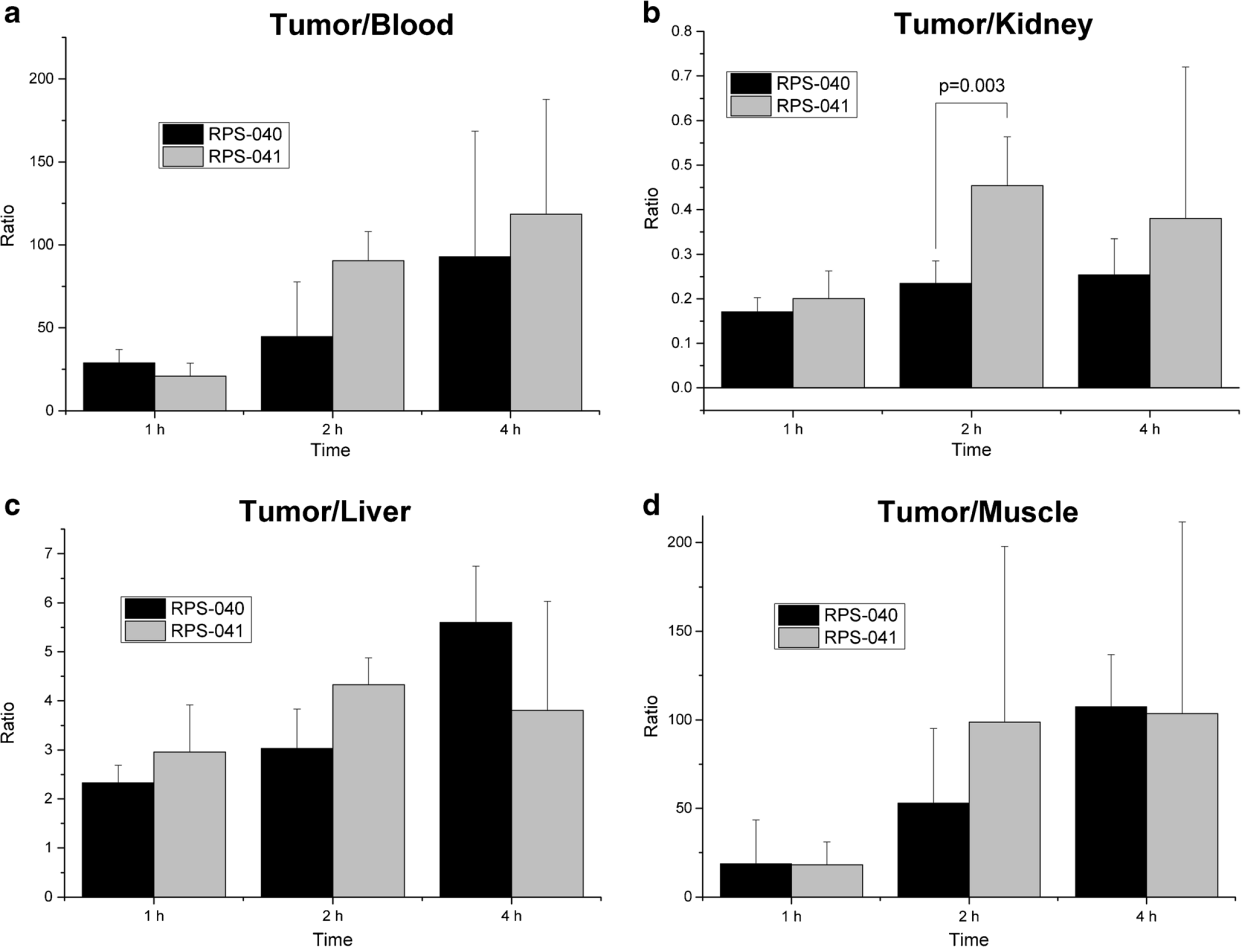
Tumor-to-background ratios of [^18^F]RPS-040 and [^18^F]RPS-041 in LNCaP xenograft tumor-bearing mice. **a** Tumor-to-blood. **b** Tumor-to-kidney. **c** Tumor-to-liver. **d** Tumor-to-muscle. The large error bars in the tumor/muscle ratio are likely to be due to the low counts recorded in the muscle

The imaging findings were corroborated in biodistribution studies where maximum tumor uptake was observed at 2 h p.i. for both ligands (Figs. 5 and 6), with [^18^F]RPS-040 reaching 14.30 ± 2.49 %ID/g (n = 5) and [^18^F]RPS-041 peaking at 10.86 ± 1.03 %ID/g (n = 5). At this time point, uptake is also observed in the kidneys (60.94 ± 8.06 and 23.93 ± 5.45), the spleen (3.23 ± 1.26 and 1.13 ± 0.47) and the liver (4.72 ± 0.93 and 2.51 ± 0.21). Tumor-to-background ratios are plotted in Fig. 7. The ratios favor [^18^F]RPS-041, which has a slightly lower tumor uptake than [^18^F]RPS-040 at all time points, but a considerably lower background signal. At 2 h p.i., contrast with [^18^F]RPS-041 is two-fold greater than with [^18^F]RPS-040.

To demonstrate that uptake was PSMA-mediated, five mice were co-injected with [^18^F]RPS-040 and 2-phosphonomethylpentanedioic acid (2-PMPA), a potent PSMA inhibitor [32], and sacrificed at 1 h p.i. At this time point, tumor uptake decreased from 12.69 ± 1.56 %ID/g (n = 5) in the unblocked set of mice to 1.75 ± 0.15 %ID/g (n = 5) in the set co-administered with 2-PMPA. Similar blocking was observed in the spleen (4.97 ± 1.55 %ID/g vs 0.32 ± 0.05 %ID/g) and kidneys (74.24 ± 10.71 %ID/g vs 5.95 ± 1.65 %ID/g; Fig. 5), two other organs known to express PSMA in nude mice [33].

### Comparison to [^68^Ga]Ga-PSMA-HBED-CC

**Fig. 8:**
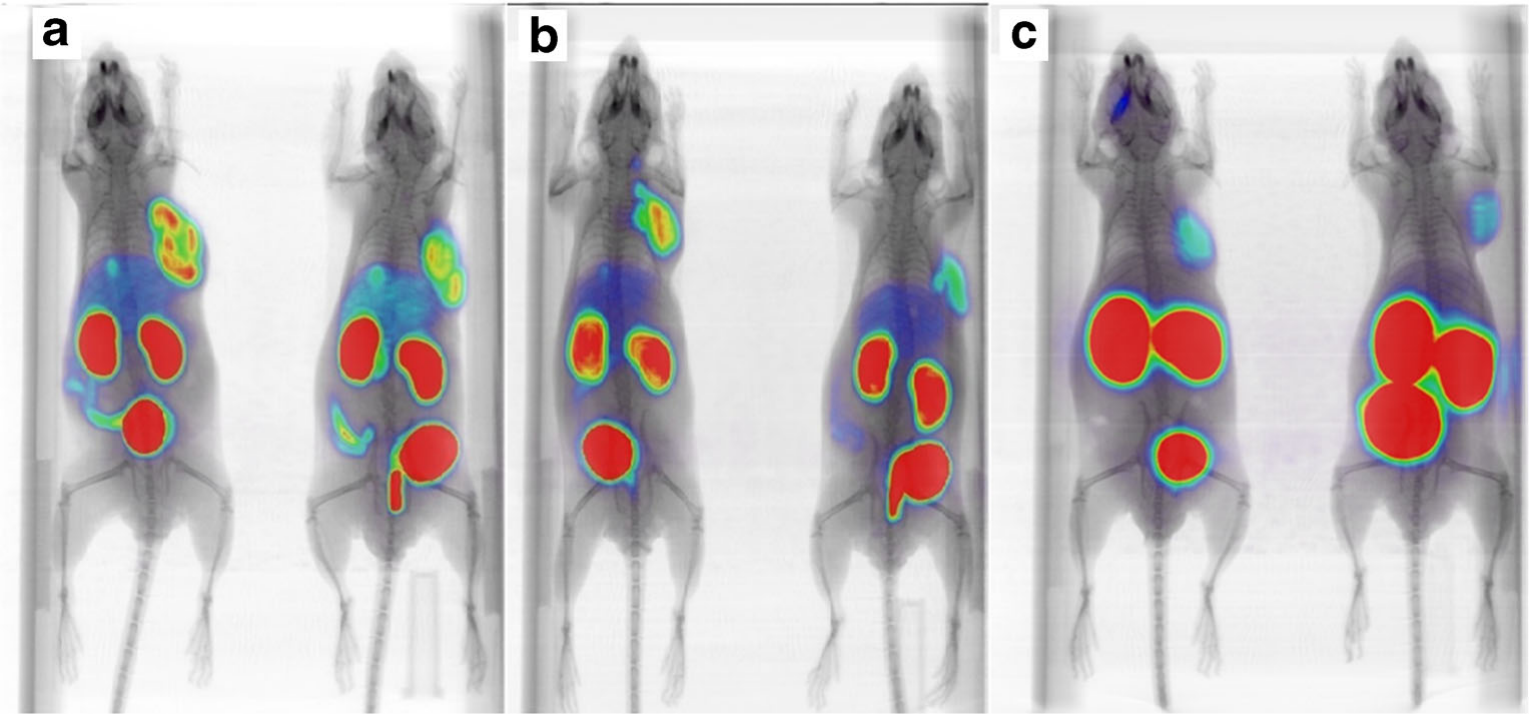
microPET/CT imaging of LNCaP xenograft tumor-bearing mice with [^18^F]RPS-040, [^18^F]RPS-041 and [^68^Ga]Ga-PSMA-HBED-CC. Mice were injected with 6.66–8.14 MBq (180–220 μCi) and imaged at 1 h p.i. **a** [^18^F]RPS-040; **b** [^18^F]RPS-041; **c** [^68^Ga]Ga-PSMA-HBED-CC

A comparison of μPET/CT images of [^18^F]RPS-040 and [^18^F]RPS-041 with [^68^Ga]Ga-PSMA-HBED-CC shows significantly higher kidney uptake (*p* < 0.0002) and lower tumor uptake (*p* < 0.0008) in the gallium-68 tracer at 1 h p.i. (Fig. 8). No uptake in the liver or intestine is evident in the [^68^Ga]Ga-PSMA-HBED-CC.

**Fig. 9:**
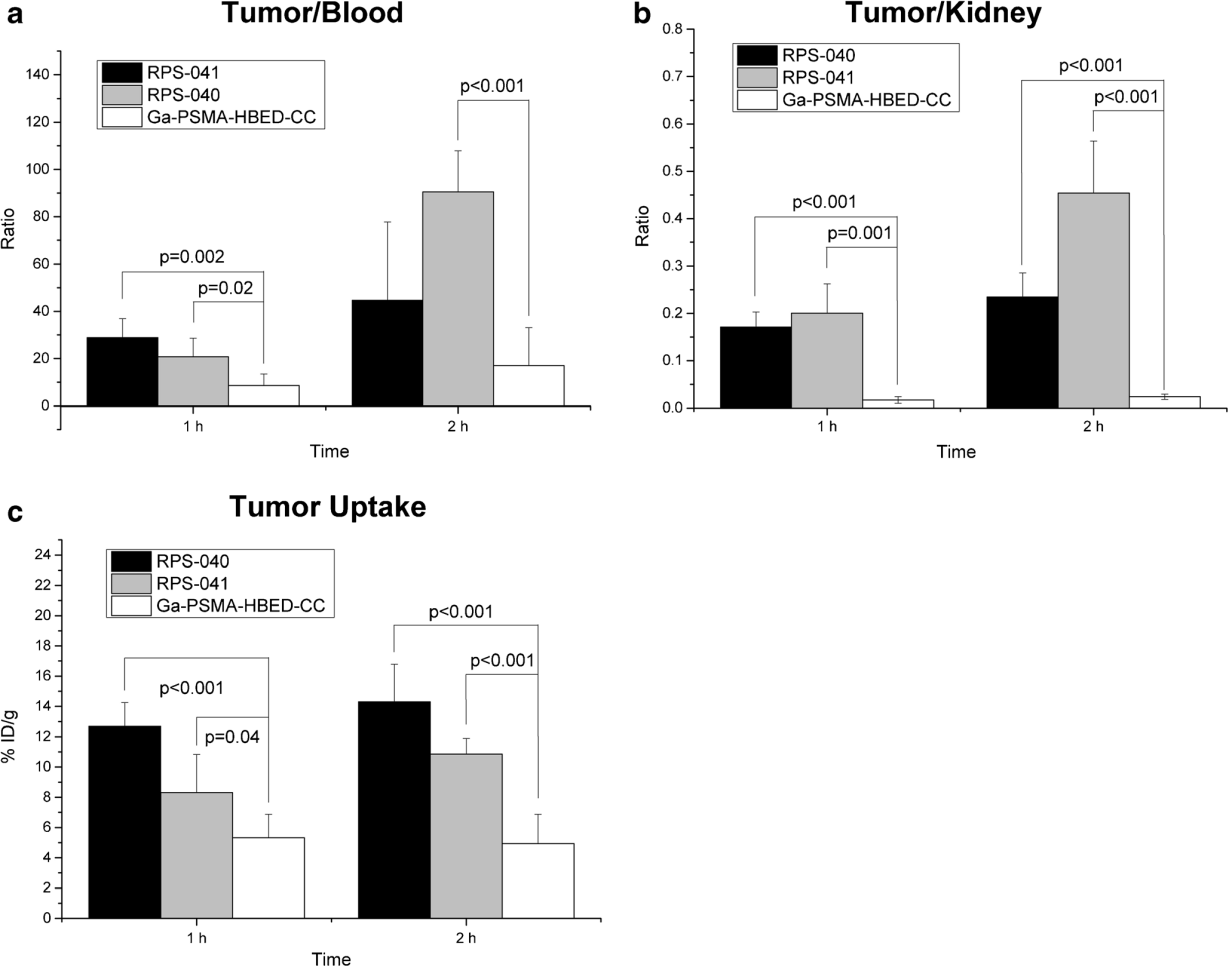
Comparison of uptake in tumor, kidney and blood between [^18^F]RPS-040, [^18^F]RPS-041 and [^68^Ga]Ga-PSMA-HBED-CC. **a** Tumor-to-blood ratio at 1 h p.i. and 2 h ([^68^Ga]Ga-PSMA-HBED-CC) or 3 h p.i. **b** Tumor-to-kidney ratio. **c** Tumor uptake

These observations are reflected in the findings of ex vivo biodistributions. A recently reported study of [^68^Ga]Ga-PSMA-HBED-CC demonstrated tumor uptake to be 5.81 ± 1.67 %ID/g at 1 h p.i. and 6.27 ± 1.44 %ID/g at 3 h p.i. [34]. At the time of maximum tumor uptake, therefore, [^18^F]RPS-040 shows more than two-fold greater uptake than [^68^Ga]Ga-PSMA-HBED-CC, while [^18^F]RPS-041 is nearly two-fold greater. In comparison to both [^18^F]RPS-040 and [^18^F]RPS-041, activity in the blood was higher, while activity in the kidney (314.44 ± 90.61 at 1 h; 207.97 ± 58.38 at 3 h) and the spleen (31.73 ± 14.09 at 1 h; 13.85 ± 3.53 at 3 h) was significantly higher (*p* < 0.0002 and *p* < 0.002, respectively). These pharmacokinetics result in higher tumor uptake and enhanced tumor-to-background ratios for [^18^F]RPS-040 and [^18^F]RPS-041 relative to [^68^Ga]Ga-PSMA-HBED-CC (Fig. 9).

At 1 h p.i., the tumor-to-blood ratio for [^18^F]RPS-041 and [^18^F]RPS-040 are more than two-fold and three-fold greater, respectively, than for [^68^Ga]Ga-PSMA-HBED-CC, and these ratios continue to grow with time. The tumor-to-kidney ratio is even more striking, as [^18^F]RPS-040 and [^18^F]RPS-041 demonstrate ten- and twelve-fold greater contrast, respectively; once again, the relative contrast increases with time.

## Discussion

A prosthetic group strategy was envisioned for the synthesis and radiosynthesis of the six PSMA inhibitors. By this approach, it was possible to overcome the interaction between fluoride and the urea protons [35] which contributes to low yield and high variability in the direct fluorination of urea-based imaging probes. Copper(I)-catalyzed click reactions have proven to be useful in radiochemistry due to their rapid kinetics and broad functional group tolerance, which allow labeling under mild conditions and in the absence of protecting groups as well as reduced reaction times [36–38]. 2-[^18^F]Fluoroethylazide has been reported to be synthesized in good radiochemical yields and to react with small molecules and peptides [27, 39–41]. It has three principal advantages in the context of radiochemical synthesis: 1) it is the smallest azide that can be radiofluorinated, and the size of the resulting [^18^F]fluoroethyltriazole permits it to be incorporated into small molecules without necessarily disrupting activity [39]; 2) it can be purified by distillation, leading to high specific activity productions [27, 39]; and 3) when Cu(I) is in stoichiometric excess, as it typically is for reactions on a radiochemical scale, the click reaction is second-order with respect to the alkyne [39, 42, 43].

The application of the 2-[^18^F]fluoroethylazide/Cu(I)-catalyzed click chemistry methodology to the radiosynthesis of small PSMA-binding molecules was demonstrated to be a straightforward and reproducible route to high-affinity ligands synthesized in good radiochemical yield. The synthesis of 2-[^18^F]fluoroethylazide was found to be highly efficient and reproducible, though substantial losses were observed during distillation. Although addition of small aliquots of MeCN to the reaction vial increased the recovery of 2-[^18^F]fluoroethylazide after distillation, the additional volume of MeCN was found to suppress the yield of the click reaction.

Optimization of the click reaction is ongoing, but early experiences have highlighted the sensitivity of the reaction to total reaction volume and the composition of reaction solvents. Conversion to the [^18^F]fluorinated triazoles was better in smaller reaction volumes; this is likely to be the consequence of higher reagent concentration. However, in reactions with similar volumes, those that had a higher MeCN content gave poorer triazole yields. This is consistent with reports of the percentage of DMF in the reaction mixture playing an important role in maintaining high levels of Cu(I) [39]. The similar boiling points of MeCN and 2-[^18^F]fluoroethylazide prevent concentration of the distillate, limiting the minimum volume of MeCN that can be used. Therefore, improvements in the yield of the click reaction will likely arise from increasing the concentration of alkyne and/or the CuSO_4_/sodium ascorbate mixture.

Recently, a potential [^18^F]fluorinated PSMA ligand synthesized by a click approach using 2-([^18^F]fluorophenyl)acetylene as a synthon has been reported [41]. The biological characteristics and pharmacokinetics of the ligand have yet to be established. Although the decay-corrected radiochemical yield was reported to be 30 %, the synthon is synthesized in 3 steps, and this limits even further the potential for automation of the process. Therefore, the use of 2-[^18^F]fluoroethylazide as the prosthetic group for radiofluorination appears to be a more promising route to a [^18^F]fluorinated PSMA ligand produced in quantities appropriate for clinical use.

The potent PSMA inhibitor MIP-1095 was used as a structural lead due to its high affinity for PSMA and high tumor uptake in both LNCaP xenograft tumor-bearing mice [44] and in humans [45]. The rigidity of the phenylurea is credited with improved potency relative to the amide analogue MIP-1072 [46] and was, therefore, a key feature retained in the structure of the newly described ligands. The ethynyl moiety has been described as a potential bioisosphere of iodine [47], and the subsequent 1,2,3-triazole has been proposed to be a bioisosphere of amide bonds [48], suggesting that the structural modifications might not have a severely adverse effect on affinity for PSMA. Furthermore, the fluoroethyl moiety might project more deeply into the S1 accessory hydrophobic pocket identified in crystal structures of glutamate carboxypeptidase II with bound ligands [49]. While the six triazolyl urea compounds ultimately displayed lower affinities for PSMA than MIP-1095, the imaging and biodistribution studies demonstrated that of the promising pharmacokinetics of the structural lead were retained.

The SAR studies highlight two clear trends in tumor uptake. Compounds in series 2 had greater uptake than their counterparts in series 1 (Table 1), indicating a preference for direct conjugation of the triazole ring to the phenylurea moiety. Derivatization of the ε-amine of Glu-urea-Lys with a rigid arm has previously been shown to improve affinity for PSMA relative to flexible linkers in a series of amide-based PSMA-targeting fluorescent probes [50] . It is likely that the lack of conformational flexibility in members of series 2 relative to members of series 1 contributes to the improvements observed in binding affinity and tumor uptake.

The second trend emerges from a comparison of the substitution position on the phenyl ring. Tumor uptake is in the order 3 > 4 > 2 for both series of compounds (Table 1). This order of preference was unexpected given previous SAR studies with a halogenated small molecule PSMA inhibitor, which indicated a strong preference for substitution at the 4-position [26], and with (alkoxyphenyl)urea derivatives of Glu-urea-Lys, for which substitution at the 2-position led to higher-affinity compounds [51]. It also did not correspond to the rank order of IC_50_ values determined in LNCaP cells. Furthermore, the two compounds substituted at the 2-position, RPS-039 and RPS-042, showed clearance via the hepatobiliary pathway in addition to renal excretion, contributing to a lower contrast tumor image. These trends suggest that substitution at the 2-position of the phenylurea does not appear to be detrimental to ligand potency as determined by in vitro assay, but it does negatively influence the in vivo imaging characteristics of the tracer.

In spite of the increased tumor uptake of the 3-substituted [^18^F]RPS-040 relative to 4-substituted [^18^F]RPS-041, the clearance of [^18^F]RPS-041 is more rapid, leading to greater image contrast and a higher tumor-to-background ratio at 2 h p.i. (Figs. 4 and 7). The clearance of [^18^F]RPS-038, the phenyl ether analogue of [^18^F]RPS-041, is similarly more rapid than [^18^F]RPS-043 (Fig. 4). It is not apparent based on the μPET/CT images alone whether the same trend is true for compounds [^18^F]RPS-043 and [^18^F]RPS-038, so the significance of the finding in the context of SAR studies requires further investigation.

The imaging characteristics of each of the six [^18^F]fluorinated triazole PSMA ligands compare favorably to [^68^Ga]Ga-PSMA-HBED-CC, the most widely used diagnostic PET imaging agent for prostate cancer. In addition to the greater sensitivity and higher spatial resolution that fluorine-18 offers over gallium-68 [52], the two- to three-fold higher tumor uptake is evident when images are compared in the same intensity scale (Fig. 8). The improved image quality is reinforced by the biodistribution studies with ligands [^18^F]RPS-040 and [^18^F]RPS-041, which showed significantly greater tumor-to-background and tumor-to-kidney ratios than [^68^Ga]Ga-PSMA-HBED-CC (Fig. 9).

[^18^F]DCFPyL, a second-generation [^18^F]fluorinated PSMA ligand currently undergoing clinical evaluation, has recently been studied in LNCaP tumor xenografts, and the maximum standardized uptake value (SUV_max_ ) was reported to be 1.1 ± 0.1 at 1 h p.i. [25]. This is lower uptake than observed for each of the six RPS ligands, for which SUV at 1 h p.i. is estimated to range from 1.5 to 2.5 and SUV_max_ in the tumor is calculated to range from approximately 1.5 to 2.9. Moreover, [^18^F]DCFPyL is reported to have a tumor-to-blood ratio of 8.3 at 1 h p.i. in LNCaP xenograft tumor-bearing mice [25]. At this same time point, the tumor-to-blood ratios for [^18^F]RPS-040 and [^18^F]RPS-041 are 28.8 ± 8.06 and 20.78 ± 7.87, respectively. By 4 h p.i., the prolonged tumor retention and rapid blood clearance drives the ratios to 92.93 ± 75.67 and 118.4 ± 69.4, respectively.

[^18^F]DCFPyL was compared by μPET/CT imaging to the RPS ligands in the same LNCaP xenograft tumor-bearing mice. Clearance from the kidneys was rapid, but rapid tumor washout was also evident (Fig. 4). These pharmacokinetics contribute to a reduction in signal in the kidneys, but also to poorer tumor delineation than can be achieved with the RPS series. The in vitro binding of PSMA-targeting ligands to mouse kidney cells was reported to be at least two-fold greater than binding to human kidney cells [53], suggesting that rapid kidney clearance in pre-clinical mouse models of prostate cancer is not an essential requirement. In this light, the greater tumor uptake, longer tumor retention and greater tumor-to-blood ratios of [^18^F]RPS-040 and [^18^F]RPS-041 compared to [^18^F]DCFPyL are favorable characteristics of these potential PET imaging agents.

Our approach to the development of PET-based PSMA tracers focused on fluorine-18 due its near optimal PET imaging characteristics [54] and the application of the radiosynthon, [^18^F]fluoroethylazide, allowed us to achieve our radiosynthetic goals simply and efficiently. The use of [^18^F]fluoroethylazide may add further complexity to automation when using single-purpose or cassette-based synthesis units, in use today by many PET centers, due to the need to label the synthon, purify it by distillation, and carry out the subsequent click reaction in a single reaction pot. Initial work has begun on the adaptation of commercially available cassettes to facilitate the distillation step. In parallel, an affordable, fit-for-purpose custom radiosynthesis box is being developed. The robust chemical and mechanical reproducibility of a synthesis box of this type has already been demonstrated by our group [55].

## Conclusions

Six PSMA inhibitors were synthesized and [^18^F]fluorinated by a Cu(I)-catalyzed click reaction involving a 2-[^18^F]fluoroethylazide prosthetic group. Although the radiochemistry is currently undergoing optimization, the initial isolation of the [^18^F]RPS series in 105 min and 20–40 % decay-corrected yield is a promising starting point for further optimization and automation. Each of the compounds showed high affinity for PSMA (4–36 nM) in LNCaP cells and good tumor uptake (>5 %ID/g) in LNCaP xenograft tumor-bearing mice. The compounds compare favorably to [^68^Ga]Ga-PSMA-HBED-CC, a PSMA imaging probe in widespread clinical development, and to [^18^F]DCFPyL, a [^18^F]fluorinated PSMA ligand currently under clinical investigation. Two ligands in particular, [^18^F]RPS-040 and [^18^F]RPS-041, show excellent tumor uptake and retention (>10 %ID/g) and high tumor-to-background and tumor-to-kidney ratios. The simple radiosynthesis, promising biological characteristics and numerous beneficial attributes of fluorine-18 as a radionuclide for imaging are evidence for the merit of further clinical development of these ligands as agents for the imaging of prostate cancer by positron emission tomography.

## Electronic supplementary material

Below is the link to the electronic supplementary material.

ESM 1 (PDF 510 kb)

## References

[1] Ferlay J, Soerjomataram I, Ervik M, Dikshit R, Eser S, Mathers C, et al. GLOBOCAN 2012: Estimated Cancer Incidence, Mortality and Prevalence Worldwide in 2012. International Agency for Research on Cancer. Available at globocan.iarc.fr/Pages/fact_sheets_cancer.aspx. Accessed on 18 May 2016.

[2] Naghavi M, Wang H, Lozano R (2015). Global, regional, and national age-sex specific all-cause and cause-specific mortality for 240 causes of death, 1990–2013: a systematic analysis for the Global Burden of Disease Study 2013. Lancet.

[3] Siegel RL, Miller KD, Jemal A (2016). Cancer Statistics, 2016. CA Cancer J Clin.

[4] National Cancer Institute Web site. SEER stat fact sheets: prostate. http://seer.cancer.gov/statfacts/html/prost.html Accessed 20 Dec 2012.

[5] Bouchelouche K, Choyke PL, Capala J (2010). Prostate specific membrane antigen – a target for imaging and therapy with radionuclides. Discov Med.

[6] Silver DA, Pellicer I, Fair WR, Heston WDW, Cordon-Cardo C (1997). Prostate-specific membrane antigen expression in normal and malignant human tissues. Clin Cancer Res.

[7] Rajasekaran SA, Anilkumar G, Oshima E, Bowie JU, Liu H, Heston W (2003). A novel cytoplasmic tail MXXXL motif mediates the internalization of prostate-specific membrane antigen. Mol Biol Cell.

[8] Ross JS, Sheehan CE, Fisher HA, Kaufman RP, Kaur P, Gray K (2003). Correlation of primary tumor prostate-specific membrane antigen expression with disease recurrence in prostate cancer. Clin Cancer Res.

[9] Bander NH, Trabusli EJ, Kostakoglu L, Yao D, Vallabhajosula S, Smith-Jones P (2003). Targeting metastatic prostate cancer with radiolabeled monoclonal antibody J591 to the extracellular domain of prostate specific membrane antigen. J Urol.

[10] Vallabhajosula S, Kuji I, Hamacher KA, Konishi S, Kostakoglu L, Kothari PA (2005). Pharmacokinetics and biodistribution of 111In- and 177Lu-labeled J591 antibody specific for prostate-specific membrane antigen: prediction of 90Y-J591 dosimetry based on 111In or 177Lu?. J Nucl Med.

[11] Srinivasarao M, Galliford CV, Low PS (2015). Principles in the design of ligand-targeted cancer therapeutics and imaging agents. Nat Rev Drug Discov.

[12] Lütje S, Heskamp S, Cornelissen AS, Poeppel TD, van den Broek SAMW, Rosenbaum-Krumme S (2015). PSMA ligands for radionuclide imaging and therapy of prostate cancer: clinical status. Theranostics.

[13] Benešová M, Schäfer M, Bauder-Wüst U, Afshar-Oromieh A, Kratochwil C, Mier W (2015). Preclinical evaluation of a tailor-made DOTA-conjugated PSMA inhibitor with optimized linker moiety for imaging and endoradiotherapy of prostate cancer. J Nucl Med.

[14] Wüstemann T, Bauder-Wüst U, Schäfer M, Eder M, Benesova M, Leotta K (2016). Design of internalizing PSMA-specific Glu-ureido-based radiotherapeuticals. Theranostics.

[15] Rahmim A, Zaidi H (2008). PET versus SPECT: strengths, limitations and challenges. Nucl Med Commun.

[16] Alavi A, Basu S (2008). Planar and SPECT imaging in the era of PET and PET-CT: can it survive the test of time. Eur J Nucl Med Mol Imaging.

[17] Cascini GL, Asabella AN, Notaristefano A, Restuccia A, Ferrari C, Rubini D, et al. 124Iodine: a longer-life positron emitter isotope – new opportunities in molecular imaging. BioMed Res. Int. 2014, Article ID 672094. 10.1155/2014/672094 PMC4034399 24895600

[18] Afshar-Oromieh A, Avtzi E, Giesel FL, Holland-Letz T, Linhart HG, Eder M (2015). The diagnostic value of PET/CT imaging with the (68)Ga-labelled PSMA ligand HBED-CC in the diagnosis of recurrent prostate cancer. Eur J Nucl Med Mol Imaging.

[19] Mease RC, Dusich CL, Foss CA, Ravert HT, Dannals RF, Seidel J (2008). N-[N-[(S)-1,3-dicarboxypropyl]carbamoyl]-4-[18F]fluorobenzyl-L-cysteine, [18F]DCFBC: a new imaging probe for prostate cancer. Clin Cancer Res.

[20] Cho SY, Gage KL, Mease RC, Senthamizhchelvan S, Holt DP, Jeffrey-Kwanisai A (2012). Biodistribution, tumor detection, and radiation dosimetry of 18F-DCFBC, a low-molecular-weight inhibitor of prostate-specific membrane antigen, in patients with metastatic prostate cancer. J Nucl Med.

[21] Rowe SP, Gage KL, Faraj SF, Macura KJ, Cornish TC, Gonzalez-Roibon N (2015). 18F-DCFBC PET/CT for PSMA-based detection and characterization of primary prostate cancer. J Nucl Med.

[22] Chen Y, Pallumbhatla M, Foss CA, Byun Y, Nimmagadda S, Senthamizhchelvan S (2011). 2-(3-{1-carboxy-5-[(6-[18F]fluoro-pyridine-3-carbonyl)-amino]-pentyl}-ureido)-pentanedioic acid, [18F]DCFPyL, a PSMA-based PET imaging agent for prostate cancer. Clin Cancer Res.

[23] Dietlein M, Kobe C, Kuhnert G, Stockter S, Fischer T, Schomäcker K (2015). Comparison of [18F]DCFPyL and [68Ga]Ga-PSMA-HBED-CC for PSMA-PET imaging in patients with relapsed prostate cancer. Mol Imaging Biol.

[24] Szabo Z, Mena E, Rowe SP, Plyku D, Nidal R, Eisenberger MA (2015). Initial evaluation of [18F]DCFPyL for prostate-specific membrane antigen (PSMA)-targeted PET imaging of prostate cancer. Mol Imaging Biol.

[25] Bouvet V, Wuest M, Jans H-S, Janzen N, Genady AR, Valliant JF (2016). Automated synthesis of [18F]DCFPyL via direct radiofluorination and validation in preclinical prostate cancer models. EJNMMI Res.

[26] Maresca KP, Hillier SM, Femia FJ, Barone DKC, Joyal JL, Zimmerman CN (2009). A Series of halogenated heterodimeric inhibitors of prostate specific membrane antigen (PSMA) as radiolabeled probes for targeting prostate cancer. J Med Chem.

[27] Glaser M, Årstad E (2007). “Click labeling” with 2-[18F]Fluoroethylazide for positron emission tomography. Bioconjug Chem.

[28] Haslop A, Wells L, Gee A, Plisson C, Long N (2014). One-pot multi-tracer synthesis of novel (18)F-labeled PET imaging agents. Mol Pharm.

[29] Amor-Coarasa A, Schoendorf M, Meckel M, Vallabhajosula S, Babich J. Comprehensive quality control of the ITG Ge-68/Ga-68 generator and synthesis of Ga-68-DOTATOC and Ga-68-PSMA-HBED-CC for clinical imaging. J Nucl Med. 2016 10.2967/jnumed.115.171249 27103024

[30] Hillier SM, Maresca KP, Lu G, Merkin RD, Marquis JC, Zimmerman CN (2013). 99mTc-labeled small-molecule inhibitors of prostate-specific membrane antigen for molecular imaging of prostate cancer. J Nucl Med.

[31] Eder M, Neels O, Müller M, Bauder-Wüst U, Rembe Y, Schäfer M (2014). Novel preclinical and radiopharmaceutical aspects of [68Ga]Ga-HBED-CC: a new PET tracer for imaging of prostate cancer. Pharmaceuticals.

[32] Jackson PF, Cole DC, Slusher BS, Stetz SL, Ross LE, Donzanti B (1996). Design, synthesis, and biological activity of a potent inhibitor of the neuorpeptidase N-acetylated α-linked acidic dipeptidase. J Med Chem.

[33] Bacich DJ, Pinto JT, Tong WP, Heston WDW (2001). Cloning, expression, genomic localization, and enzymatic activities of the mouse homolog of prostate-specific membrane antigen/NAALADase/folate hydrolase. Mamm Genome.

[34] Nikolopoulou A, Amor-Coarasa A, Kelly J, Vallabhajosula V, Babich J (2015). Comparative evaluation of 68Ga-labeled urea-based PSMA ligands in LNCaP tumor bearing mice. J Nucl Med.

[35] Boiocchi M, Del Boca L, Gómez DE, Fabbrizzi L, Licchelli M, Monzani E (2004). Nature of urea-fluoride interaction: incipient and definitive proton transfer. J Am Chem Soc.

[36] Walsh JC, Kolb HC (2010). Applications of click chemistry in radiopharmaceutical development. Chimia.

[37] Glaser M, Robins EG (2009). ‘Click labelling’ in PET radiochemistry. J Label Compd Radiopharm.

[38] Nwe K, Brechbiel MW (2009). Growing applications of “Click Chemistry” for bioconjugation in contemporary biomedical research. Cancer Biother Radiopharm.

[39] Zhou D, Chu W, Dence CS, Mach RH, Welch MJ (2012). Highly efficient click labeling using 2-[18F]fluoroethyl azide and synthesis of an 18FN-hydroxysuccinimide ester as conjugation agent. Nucl Med Biol.

[40] Kotbus D, Giesen Y, Ullrich R, Backes H, Neumaier B (2009). A fully automated two-step synthesis of an F-18 labelled tyrosine kinase inhibitor for EGFR kinase activity imaging in tumors. Appl Radiat Isot.

[41] Krapf P, Richarz R, Urusova EA, Neumaier B, Zlatopolskiy BD (2016). Seyforth-gilbert homologation as a route to 18F-labeled building blocks: preparation of radiofluorinated phenylacetylenes and their application in PET chemistry. Eur J Org Chem.

[42] Rodionov VO, Fokin VV, Finn MG (2005). Mechanism of the ligand-free cui-catalyzed azide-alkyne cycloaddition reaction. Angew Chem Int Ed.

[43] Bock VD, Hiemstra H, van Maarseven JH. CuI-Catalyzed alkyne-azide “Click” cycloadditions from a mechanistic and synthetic perpective. Eur J Org Chem. 2006, 51–68.

[44] Hillier SM, Maresca KP, Femia FJ, Marquis JC, Foss CA, Nguyen N (2009). Preclinical evaluation of novel glutamate-urea-lysine analogues that target prostate-specific membrane antigen as molecular imaging pharmaceuticals for prostate cancer. Cancer Res.

[45] Zechmann CM, Afshar-Oromieh A, Armor T, Stubbs JB, Mier W, Hadaschik B (2014). Radiation dosimetry and first therapy results with a 124I/ 131I-labeled small molecule (MIP-1095) targeting PSMA for prostate cancer therapy. Eur J Nucl Med Mol Imaging.

[46] Machulkin AE, Ivanenkov YA, Aladinskaya AV, Veselov MS, Aladinskiy VA, Beloglazkina EK (2016). Small-molecule PSMA ligands. Current state, SAR and perspectives. J Drug Target.

[47] Wilcken R, Zimmerman MO, Bauer MR, Rutherford TJ, Fersht AR, Joerger AC (2015). Experimental and theoretical evaluation of the ethynyl moiety as a halogen bioisostere. ACS Chem Biol.

[48] Bock VD, Speijer D, Hiemstra H, van Maarsveen JH (2007). 1,2,3-Triazoles as peptide bond isosteres: synthesis and biological evaluation of cyclotetrapeptide mimics. Org Biomol Chem.

[49] Barinka C, Byun Y, Dusich CL, Banerjee SR, Chen Y, Castanares M (2008). Interactions between human glutamate carboxypeptidase II and urea-based Inhibitors: structural characterization. J Med Chem.

[50] Tykvart J, Schimer J, Jančařík A, Bařinková J, Navrátil V, Starková J (2015). Design of Highly Potent Urea-Based, Exosite-Binding Inhibitors Selective for Glutamate Carboxypeptidase II. J Med Chem.

[51] Tykvart J, Schimer J, Bařinková J, Pachl P, Poštová-Slavětínská L, Majer P (2014). Rational design of urea-based glutamate carboxypeptidase II (GCPII) inhibitors as versatile tools for specific drug targeting and delivery. Bioorg Med Chem.

[52] Sanchez-Crespo A (2013). Comparison of gallium-68 and fluorine-18 imaging characteristics in positron emission tomography. Appl Radiat Isot.

[53] Chatalic KLS, Heskamp S, Konijnenberg M, Molkenboer-Kuenen JDM, Franssen GM, Clahsen-van Groningen MC (2016). Towards personalized treatment of prostate cancer: PSMA I&T, a promising prostate-specific membrane antigen-targeted theranostic agent. Theranostics.

[54] Mittra E, Quon A (2009). Positron emission tomography/computed tomography: the current technology and applications. Radiol Clin N Am.

[55] Amor-Coarasa A, Kelly JM, Babich JW (2015). Synthesis of [^11^C]palmitic acid for PET imaging using a single molecular sieve 13X cartridge for reagent trapping, radiolabeling and selective purification. Nucl Med Biol.

