# Supplementary material for: Synthesis and pre-clinical evaluation of a new class of high-affinity 18F-labeled PSMA ligands for detection of prostate cancer by PET imaging
Source: Eur J Nucl Med Mol Imaging. 2016 Nov 15;44(4):647–61. doi: 10.1007/s00259-016-3556-5 (PMC5323493; doi:10.1007/s00259-016-3556-5)
Supplement: Supplementary file 1 — (PDF 510 kb) [file 259_2016_3556_MOESM1_ESM.pdf]

SUPPLEMENTARY INFORMATION FOR

Synthesis and Pre-Clinical Evaluation of a New Class of High Affinity ^{18}F-Labeled PSMA Ligands for Detection of Prostate Cancer by PET Imaging

James Kelly[1], Alejandro Amor-Coarasa[1], Anastasia Nikolopoulou[1,2], Dohyun Kim[2], Clarence Williams Jr[1], Shashikanth Ponnala[1], John W. Babich[1,2,3]

[1]Division of Radiopharmaceutical Sciences and Molecular Imaging Innovations Institute, Department of Radiology, Weill Cornell Medicine, New York, NY 10021

[2]Citigroup Biomedical Imaging Center, Weill Cornell Medicine, New York, NY 10021

[3]Meyer Cancer Center, Weill Cornell Medicine, New York, NY 10021

Corresponding author:

Professor John W Babich PhD

Department of Radiology

Weill Cornell Medicine

Belfer Research Building, Room 1600

413 East 69th Street

New York, NY 10021

Office: +1-646-962-6148; Fax: +1-646-962-0577

Email: job2060@med.cornell.edu

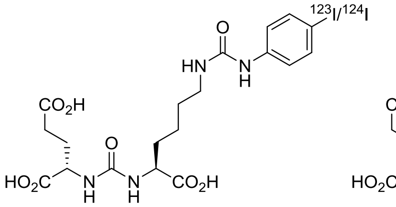

^{123}I/^{124}I-MIP-1095

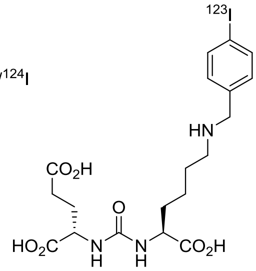

^{123}I-MIP-1072

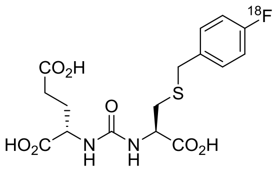

[^{18}F]DCFBC

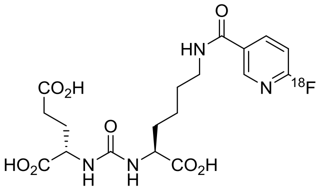

[^{18}F]DCFPyL

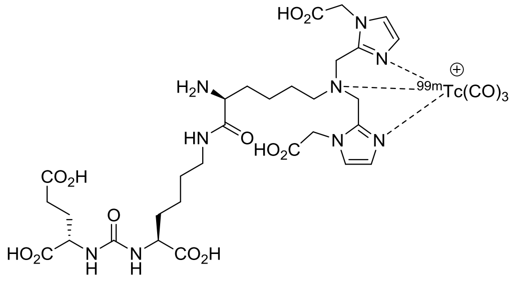

^{99m}Tc-MIP-1405

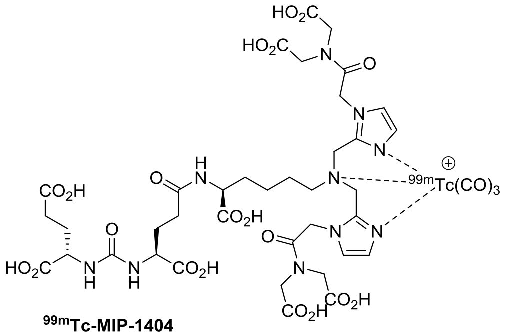

^{99m}Tc-MIP-1404

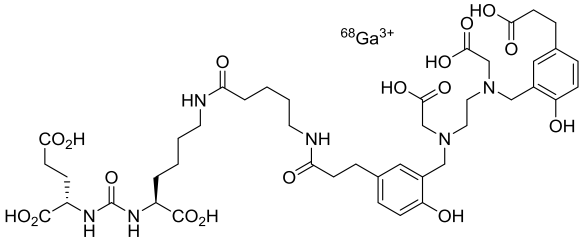

[^{68}Ga]Ga-PSMA-HBED-CC

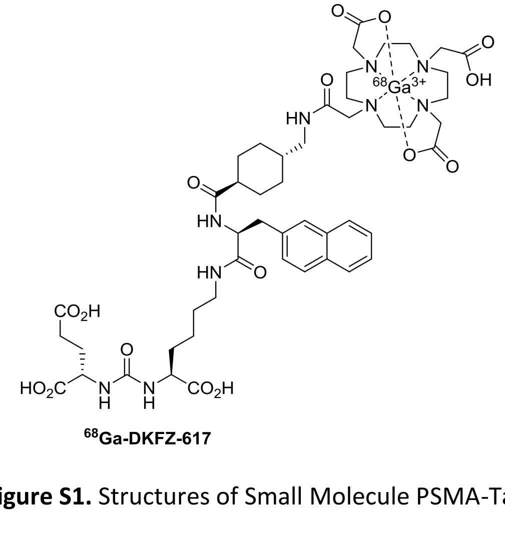

^{68}Ga-DKFZ-617

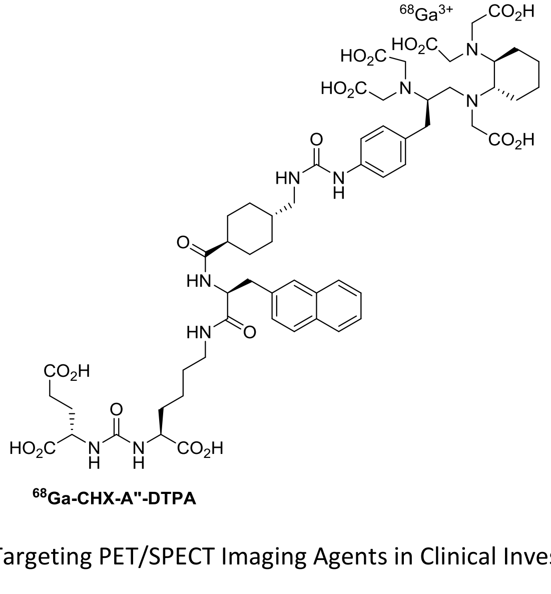

^{68}Ga-CHX-A"-DTPA

Figure S1. Structures of Small Molecule PSMA-Targeting PET/SPECT Imaging Agents in Clinical Investigation

Compound Synthesis

General Methods

All solvents were purchased from Sigma Aldrich and were of reagent grade quality unless otherwise indicated. Solvents were dried either by distillation over an activated stainless steel column (Pure Process Technology, LLC) column or by drying over activated molecular sieves. Reagents were purchased from Sigma Aldrich, Alfa Aesar, Combi Blocks, ChemBridge and Enamine, and were of reagent grade with the exception of 3-(prop-2-yn-1-yloxy)aniline (Enamine), which was 80-85% pure by HPLC.

All reactions described below were carried out in dried glassware. Purifications were performed using silica chromatography on VWR® High Purity Silica Gel 60 Å. Preparative HPLC was performed using an XBridge™ Prep C18 5 µm OBD™ 19 x 100 mm column (Waters) on a dual pump Agilent ProStar HPLC fitted with an Agilent ProStar 325 Dual Wavelength UV-Vis Detector. UV absorption was monitored at 220 nm and 280 nm. A binary solvent system was used, with solvent A comprising H_2O + 0.01% TFA and solvent B consisting of 90% v/v MeCN/H_2O + 0.01% TFA. Purification was achieved using the following gradient HPLC method: 0%B 0-1 min., 0-100%B 1-28 mins., 100-0%B 28-30 mins.

Final products were identified and characterized using thin layer chromatography, analytical HPLC, mass spectroscopy and NMR spectroscopy. Analytical HPLC was performed using an XSelect™ CSH™ C18 5 µm 4.6 x 50 mm column (Waters). Mass determinations were performed by LCMS analysis using a Waters ACQUITY UPLC® coupled to a Waters SQ Detector 2. NMR analyses were performed using a Bruker Avance III 500 MHz spectrometer. Spectra are reported as ppm and are referenced to the solvent resonances in in DMSO-d6 or chloroform-d (Sigma Aldrich). The purity of all compounds evaluated in the biological assay was > 95% purity as judged by LC-MS and 1H NMR.

Route A Synthesis

Di-*tert*-butyl (((*S*)-1-(*tert*-butoxy)-6-(1*H*-imidazole-1-carboxamido)-1-oxohexan-2-yl)carbamoyl)-*L*-glutamate (2)

Compound **1** (1.46 g, 3.0 mmol) was dissolved in dichloroethane (10 mL) with triethylamine (0.84 mL, 6.0 mmol) and a catalytic amount of *N*,*N*'-dimethylaminopyridine (15 mg) and stirred at room temperature under Ar. After 5 min, a suspension of 1,1'-carbonyldiimidazole (486 mg, 3.3 mmol) in dichloroethane (2 mL) was added, and the reaction was stirred overnight under Ar. The solution was then washed successively with 1% v/v AcOH in H_2O and saturated NaCl solution. The organic layer was dried over $MgSO_4$, filtered and concentrated under reduced pressure to give a yellow oil. The oil was

purified by silica chromatography (50% EtOAc in hexanes to 10% MeOH in EtOAc) to give the product, di-*tert*-butyl (((*S*)-1-(*tert*-butoxy)-6-(1*H*-imidazole-1-carboxamido)-1-oxohexan-2-yl)carbamoyl)-*L*-glutamate (**2**), as an off white powder (60% yield). ^{1}H NMR (500 MHz, $CDCl_3$) δ 8.34 (s, 1H), 7.94 (br s, 1H), 7.69 (s, 1H), 7.05 (s, 1H), 5.95 (d, 1H, J = 7.8 Hz), 5.58 (d, 1H, J = 7.6 Hz), 4.21 (m, 1H), 4.16 (m, 1H), 3.53 (m, 1H), 3.28 (m, 1H), 2.30 (m, 2H), 2.05 (m, 1H), 1.83 (m, 1H), 1.79 (m, 1H), 1.72 (m, 1H), 1.50 (m, 2H), 1.43 (s, 18H), 1.38 (s, 9H), 1.32 (m, 2H). ESI(+) = 582.5 $(M+H)^+$. Calculated mass: 581.34

Di-*tert*-butyl (((*S*)-1-(*tert*-butoxy)-1-oxo-6-(3-(2-prop-2-yn-1-yloxy)phenyl)ureido)hexan-2-yl)carbamoyl)-*L*-glutamate (3)

A solution of di-*tert*-butyl (((*S*)-1-(*tert*-butoxy)-6-(1*H*-imidazole-1-carboxamido)-1-oxohexan-2-yl)carbamoyl)-*L*-glutamate (**2**) (182 mg, 0.30 mmol) in dichloroethane (4 mL) was cooled to 0°C and stirred under Ar. A solution of triethylamine (87 µL, 0.63 mmol) in dichloroethane (1 mL) was added followed by a solution of methyl triflate (34 uL, 0.31 mmol) in dichloroethane (1 mL). The reaction was stirred for 60 min, warming to room temperature. Then 2 mL of the reaction mixture was transferred under Ar to a round-bottom flask containing a solution of 2-(2-propyn-1-yloxy)aniline (15 mg, 0.10 mmol) in dichloroethane (1 mL). The resulting mixture was stirred at room temperature for 16 h under Ar. The mixture was then cooled to room temperature and concentrated under reduced pressure to give an oil. The oil was purified by reverse phase prep HPLC (12 mL/min, 0% B to 100% B over 30 min followed by 5 min at 100% B; λ = 220 nm, 254 nm). The peak containing the product was lyophilized and the product, di-*tert*-butyl (((*S*)-1-(*tert*-butoxy)-1-oxo-6-(3-(2-prop-2-yn-1-yloxy)phenyl)ureido)hexan-2-yl)carbamoyl)-*L*-glutamate (**3**), was isolated as a white powder (28 mg, 45% yield). ^{1}H NMR (500 MHz, $CDCl_3$) δ 8.20 (d, 1H, J = 7.8 Hz), 7.45 (br s, 1H), 6.97 (m, 1H), 6.84 (m, 2H), 6.71 (m, 2H), 6.00 (br s, 1H), 5.69 (br s, 1H), 5.50 (d, 1H, J = 7.0 Hz), 4.67 (dd, 2H, J_1 = 7.2 Hz, J_2 = 2.4 Hz), 4.36 (m, 1H), 4.21 (m, 1H), 3.12 (m, 2H), 2.54 (t, 1H, J = 2.4 Hz), 2.33 (m, 2H), 2.03 (m, 1H), 1.87 (m, 1H), 1.75 (m, 1H), 1.54-1.38 (m, 5H), 1.41 (s, 18H), 1.37 (s, 9H). ESI(+) = 661.5 $(M+H)^+$. Calculated mass: 660.37

Di-*tert*-butyl (((*S*)-1-(*tert*-butoxy)-6-(3-(2-ethynylphenyl)ureido)-1-oxohexan-2-yl)carbamoyl)-*L*-glutamate (4)

The compound was synthesized by the same method from 3-ethynyl aniline (1.1 eq) and urea (**2**) (1.0 eq) and isolated as an orange semi-solid (33%). ^{1}H NMR (500 MHz, $CDCl_3$) δ 7.90 (s, 1H), 7.58 (t, 1H, J = 1.7 Hz), 7.51 (dd, 1H, J_1 = 8.2 Hz, J_2 = 1.3 Hz), 7.18 (t, 1H, J = 7.9 Hz), 7.05 (d, 1H, J = 7.7 Hz), 6.38 (d, 1H, J = 7.9 Hz), 6.28 (br s, 1H), 5.77 (d, 1H, J = 6.9 Hz), 4.32 (m, 1H), 4.02 (m, 1H), 3.53 (m, 1H), 3.05 (m, 1H),

3.00 (s, 1H), 2.39 (m, 2H), 2.07 (m, 1H), 1.88 (m, 1H), 1.74 (m, 1H), 1.62 (m, 1H), 1.49-1.37 (m, 4H), 1.41 (s, 18H), 1.37 (s, 9H). ESI(+) = 631.5 $(M+H)^+$. Calculated mass: 630.36

Di-*tert*-butyl (((*S*)-1-(*tert*-butoxy)-1-oxo-6-(3-(4-prop-2-yn-1-yloxy)phenyl)ureido)hexan-2-yl)carbamoyl)-*L*-glutamate (5)

The compound was synthesized by the same method from [4-(2-propyn-1-yloxy)phenyl]amine hydrochloride (1.1 eq) and urea (**2**) (1.0 eq) and isolated as a light brown oil (46%). ^{1}H NMR (500 MHz, $CDCl_3$) δ 7.60 (s, 1H), 7.33 (d, 2H, J = 9.0 Hz), 6.86 (d, 2H, J = 9.0 Hz), 6.24 (d, 1H, J = 7.8 Hz), 6.05 (br s, 1H), 5.71 (d, 1H, J = 7.0 Hz), 4.61 (d, 2H, J = 2.3 Hz), 4.30 (m, 1H), 4.03 (m, 1H), 3.45 (m, 1H), 3.05 (m, 1H), 2.47 (t, 1H, J = 2.3 Hz), 2.31 (m, 2H), 2.06 (m, 1H), 1.83 (m, 1H), 1.75 (m, 1H), 1.48 (m, 3H), 1.41 (s, 9H), 1,39 (s, 9H), 1.37 (s, 9H),1.31 (m, 2H). ESI(+) = 661.4 $(M+H)^+$. Calculated mass: 660.37

(((*S*)-1-Carboxy-5-(3-(2-(prop-2-yn-1-yloxy)phenyl)ureido)pentyl)carbamoyl)-*L*-glutamic acid (6)

Di-*tert*-butyl (((*S*)-1-(*tert*-butoxy)-1-oxo-6-(3-(2-prop-2-yn-1-yloxy)phenyl)ureido)hexan-2-yl)carbamoyl)-*L*-glutamate (**3**) (4.2 mg, 6.4 µmol) was dissolved in CH_2Cl_2 (0.5 mL). Trifluoroacetic acid (0.5 mL) was added, and the mixture was stirred overnight at room temperature. The volatile solvents were removed under a stream of N_2, and the resulting crude residue was lyophilized to give the product, (((*S*)-1-carboxy-5-(3-(2-(prop-2-yn-1-yloxy)phenyl)ureido)pentyl)carbamoyl)-*L*-glutamic acid (**6**) as a white powder (3.1 mg, 98% yield). ^{1}H NMR (500 MHz, DMSO-d6) δ 8.10 (m, 1H), 7.83 (s, 1H), 7.03 (m, 1H), 6.90 (br s, 1H), 6.86 (m, 2H), 6.33 (d, 1H, J = 12.5 Hz), 6.31 (d, 1H, J = 12.5 Hz), 4.86 (d, 2H, J = 2.3 Hz), 4.10 (m, 2H), 3.60 (t, 1H, J = 2.3 Hz), 3.06 (m, 2H), 2.24 (m, 2H), 1.93 (m, 1H), 1.69 (m, 2H), 1.56 (m, 1H), 1.42 (m, 2H), 1.32 (m, 2H). ESI(+) = 493.3 $(M+H)^+$. Calculated mass: 492.19

(((S)-1-carboxy-5-(3-(3-ethynylphenyl)ureido)pentyl)carbamoyl)-L-glutamic acid (7)

Alkyne (**4**) was deprotected by the same method and the title compound was isolated as a white powder (61%). ^{1}H NMR (500 MHz, DMSO-d6) δ 8.42 (s, 1H), 7.11 (m, 2H), 6.91 (d, 1H, J = 8.2 Hz), 6.50 (dd, 1H, J_1 = 8.2 Hz, J_2 = 2.4 Hz), 6.31 (m, 2H), 6.13 (br s, 1H), 4.71 (d, 2H, J = 2.2 Hz), 4.08 (m, 2H), 3.05 (m, 2H), 2.24 (m, 2H), 1.91 (m, 1H), 1.70 (m, 2H), 1.54 (m, 1H), 1.41 (m, 2H), 1.30 (m, 2H). ESI(+) = 463.3 $(M+H)^+$. Calculated mass: 462.18

((1-Carboxy-5-(3-(4-(prop-2-yn-1-yloxy)phenyl)ureido)pentyl)carbamoyl)glutamic acid (8)

Alkyne (**5**) was deprotected by the same method and the title compound was isolated as a white powder (96%). ^{1}H NMR (500 MHz, DMSO-d6) δ 8.28 (s, 1H), 7.31 (d, 2H, J = 9.0 Hz), 6.87 (d, 2H, J = 9.0 Hz), 6.35

(d, 1H, J = 11.4 Hz), 6.34 (d, 1H, J = 11.4 Hz), 6.09 (br s, 1H), 4.72 (d, 2H, J = 2.3 Hz), 4.10 (m, 2H), 3.54 (t, 1H, J = 2.3 Hz), 3.06 (m, 2H), 2.25 (m, 2H), 1.93 (m, 1H), 1.63 (m, 2H), 1.53 (m, 1H), 1.42 (m, 2H), 1.31 (m, 2H). ESI(+) = 493.3 $(M+H)^+$. Calculated mass: 492.19

Route B Synthesis

(((*S*)-5-Amino-1-carboxypentyl)carbamoyl)-*L*-glutamic acid (9)

Compound (**1**) (1.22 g, 2.5 mmol) was dissolved in CH_2Cl_2 (5 mL). Trifluoroacetic acid (1.5 mL) was added, and the reaction was stirred overnight at room temperature. The volatile materials were removed under a stream of N_2, and the crude product was lyophilized to give (((*S*)-amino-1-carboxypentyl)carbamoyl)-*L*-glutamic acid (**9**) as a viscous oil (700 mg, 88%). ^{1}H NMR (500 MHz, DMSO-d6) δ 7.71 (s, 2H), 6.37 (m, 2H), 4.08 (m, 2H), 2.78 (m, 2H), 2.25 (m, 2H), 1.93 (m, 1H), 1.70 (m, 2H), 1.53 (m, 3H), 1.32 (m, 2H).

(((S)-1-Carboxy-5-(3-(2-ethynylphenyl)ureido)pentyl)carbamoyl)-L-glutamic acid (10)

A solution of 2-ethynyl aniline (30 µL, 0.26 mmol) in toluene (1 mL) was added slowly to a solution of triphosgene (56 mg, 0.19 mmol) in toluene (3 mL) at room temperature under Ar. Triethylamine (42 µL, 0.30 mmol) was added and the reaction was heated to reflux for 6 h. The solvent was removed under reduced pressure, and the crude residue, a yellow/white semisolid, was dissolved in DMF (2 mL). Then a solution of amine (**9**) (60 mg, 0.19 mmol) in DMF (1 mL) was added, followed by triethylamine (42 µL, 0.30 mmol). The reaction was stirred at room temperature for 90 min. The mixture was concentrated under reduced pressure and the crude residue was purified by reverse phase prep HPLC (12 mL/min, 0% B to 100% B over 30 min followed by 5 min at 100% B; λ = 220 nm, 254 nm). The peak containing the product was collected and lyophilized to give (((*S*)-1-carboxy-5-(3-(2-ethynylphenyl)ureido)pentyl)carbamoyl)-*L*-glutamic acid (**10**) as a white powder (27 mg, 31% yield). ^{1}H NMR (500 MHz, DMSO-d6) δ 8.13 (d, 1H, J = 8.5 Hz), 7.86 (s, 1H), 7.37 (dd, 1H, J_1 = 7.6 Hz, J_2 = 1.3 Hz), 7.27 (m, 1H), 7.23 (br s, 1H), 6.90 (t, 1H, J = 7.6 Hz), 6.34 (m, 2H), 4.56 (s, 1H), 4.10 (m, 2H), 3.08 (m, 2H), 2.25 (m, 2H), 1.93 (m, 1H), 1.70 (m, 2H), 1.57 (m, 1H), 1.44 (m, 2H), 1.33 (m, 2H). ESI(+) = 463.5 $(M+H)^+$; ESI(-) = 461.2 $(M-H)^-$. Calculated mass: 462.18

((1-Carboxy-5-(3-(3-(prop-2-yn-1-yloxy)phenyl)ureido)pentyl)carbamoyl)glutamic acid (11)

The compound was synthesized by the same method from amine (**9**) and 3-(prop-2-yn-1-yloxy)aniline and isolated as a light brown powder (13%). ^{1}H NMR (500 MHz, DMSO-d6) δ 8.54 (s, 1H), 7.61 (s, 1H), 7.32 (dd, 1H, J_1 = 8.1 Hz, J_2 = 1.3 Hz), 7.21 (t, 1H, J = 7.8 Hz), 6.98 (d, 1H, J = 7.6 Hz), 6.33 (m, 2H), 6.21 (br

s, 1H), 4.11 (s, 2H), 4.08 (m, 2H), 3.06 (m, 2H), 2.24 (m, 2H), 1.93 (m, 1H), 1.71 (m, 2H), 1.55 (m, 1H), 1.43 (m, 2H), 1.31 (m, 2H). ESI(+) = 493.1 $(M+H)^+$. Calculated mass: 492.19

(((S)-1-carboxy-5-(3-(4-ethynylphenyl)ureido)pentyl)carbamoyl)-L-glutamic acid (12)

The compound was synthesized by the same method from amine (**9**) and 4-ethynyl aniline and isolated as a pale green powder (38%). ^1H NMR (500 MHz, DMSO-d6) δ 8.64 (s, 1H), 7.40 (d, 2H, J = 8.5 Hz), 7.32 (d, 2H, J = 8.5 Hz), 6.33 (m, 2H), 6.22 (br s, 1H), 4.10 (m, 2H), 3.99 (s, 1H), 3.07 (m, 2H), 2.25 (m, 2H), 1.93 (m, 1H), 1.70 (m, 2H), 1.55 (m, 1H), 1.43 (m, 2H), 1.31 (m, 2H). ESI(+) = 463.4 $(M+H)^+$; ESI(-) = 461.3 $(M-H)^-$. Calculated mass: 462.18

Synthesis of the triazoles

2-Fluoroethyltosylate (13)

A solution of tetrabutylammonium fluoride (2.2 mL, 1.0M in THF) was added to a suspension of di(*p*-toluenesulfonyl)ethanediol (740 mg, 2.0 mmol) in THF (15 mL), and the mixture was heated to reflux under Ar overnight. Then the reaction was cooled to room temperature and the solvent was removed under reduced pressure. The crude residue was partitioned between H_2O and EtOAc. The layers were separated and the aqueous layer was extracted with EtOAc. The organic layers were combined and dried over $MgSO_4$, filtered and concentrated under reduced pressure to give a colorless oil. The oil was purified by silica chromatography (20% EtOAc in hexanes) to give 2-fluoroethyltosylate (**13**) as a colorless oil (225 mg, 52% yield). ^1H NMR (500 MHz, $CDCl_3$) δ 7.79 (d, 2H, J = 8.5 Hz), 7.35 (d, 2H, J = 8.6 Hz), 4.61 (m, 1H), 4.51 (m, 1H), 4.28 (m, 1H), 4.22 (m, 1H), 2.45 (s, 3H).

(((*S*)-1-Carboxy-5-(3-(2-(1-(2-fluoroethyl)-1*H*-1,2,3-triazol-4-yl)phenyl)ureido)pentyl)carbamoyl)-*L*-glutamic acid (RPS-042)

Sodium azide (10 mg, 150 µmol) was suspended in a solution of 2-fluoroethyltosylate (7.5 mg, 30 µmol) in DMF (0.3 mL). The suspension was stirred overnight at room temperature and then filtered. To the filtrate was added a solution of alkyne (**6**) (0.9 mg, 1.83 µmol) in DMSO (0.2 mL). In a separate vial, 0.5M $CuSO_4$ (100 µL) and 1.5M sodium ascorbate (100 µL) were mixed for 5 min and then transferred to the reaction vial as a solution in DMF (100 µL). The reaction was stirred for 60 min at room temperature and was then purified by reverse phase prep HPLC (12 mL/min, 0% B to 100% B over 30 min followed by 5 min at 100% B; λ = 220 nm, 254 nm). The peak containing the product was lyophilized, and RPS-042 was isolated as a white powder (0.8 mg, 75% yield). ^1H NMR (500 MHz, DMSO-d6) δ 8.64 (s, 1H), 8.16 (d, 1H,

J = 8.1 Hz), 7.61 (d, 1H, J = 7.8 Hz), 7.26 (dd, 1H, J_1 = 8.3 Hz, J_2 = 7.3 Hz), 7.12 (br s, 1H), 7.02 (m, 2H), 6.33 (d, 1H, J = 8.3 Hz), 6.31 (d, 1H, J = 8.4 Hz), 4.97 (m, 1H), 4.86 (m, 2H), 4.80 (m, 1H), 4.10 (m, 2H), 3.07 (m, 2H), 2.25 (m, 2H), 1.94 (m, 1H), 1.70 (m, 2H), 1.57 (m, 1H), 1.44 (m, 2H), 1.32 (m, 2H). ESI(+) = 552.4 $(M+H)^+$; ESI(-) = 550.3 $(M-H)^-$. Calculated mass: 551.21

(((*S*)-1-Carboxy-5-(3-(3-(1-(2-fluoroethyl)-1*H*-1,2,3-triazol-4-yl)phenyl)ureido)pentyl)carbamoyl)-*L*-glutamic acid (RPS-040)

RPS-040 was synthesized from alkyne (**7**) by the same method and isolated as a white powder (82% yield). ^{1}H NMR (500 MHz, DMSO-d6) δ 8.52 (s, 2H), 7.94 (s, 1H), 7.32 (m, 2H), 7.26 (m, 1H), 6.32 (m, 2H), 6.15 (t, 1H, J = 5.2 Hz), 4.91 (t, 1H, J = 4.6 Hz), 4.82 (t, 1H, J = 4.6 Hz), 4.77 (t, 1H, J = 4.6 Hz), 4.71 (t, 1H, J = 4.6 Hz), 4.08 (m, 2H), 3.07 (d, 2H, J_1 = 12.4 Hz, J_2 = 6.8 Hz), 2.25 (m, 2H), 1.91 (m, 1H), 1.67 (m, 2H), 1.54 (m, 1H), 1.43 (m, 2H), 1.30 (m, 2H). ESI(+) = 552.4 $(M+H)^+$. ESI(-) = 550.3. Calculated mass: 551.21

(((*S*)-1-Carboxy-5-(3-(4-(1-(2-fluoroethyl)-1*H*-1,2,3-triazol-4-yl)phenyl)ureido)pentyl)carbamoyl)-*L*-glutamic acid (RPS-041)

RPS-041 was synthesized from alkyne (**8**) by the same method and isolated as a white powder (50% yield). ^{1}H NMR (500 MHz, DMSO-d6) δ 8.57 (s, 1H), 8.48 (s, 1H), 7.70 (d, 2H, J = 8.6 Hz), 7.47 (d, 2H, J = 8.6 Hz), 6.35 (d, 1H, J = 9.3 Hz), 6.33 (d, 1H, J = 9.3 Hz), 6.22 (br s, 1H), 4.92 (t, 1H, J = 4.7 Hz), 4.83 (t, 1H, J = 4.7 Hz), 4.77 (t, 1H, J = 4.7 Hz), 4.72 (t, 1H, J = Hz), 4.10 (m, 2H), 3.10 (m, 2H), 2.21 (m, 2H), 1.93 (m, 1H), 1.85 (m, 2H), 1.63 (m, 1H), 1.43 (m, 2H), 1.33 (m, 2H). ESI(+) = 552.5 $(M+H)^+$; ESI(-) = 550.3 $(M-H)^-$. Calculated mass: 551.21

(((*S*)-1-Carboxy-5-(3-(2-((1-(2-fluoroethyl)-1*H*-1,2,3-triazol-4-yl)methoxy)phenyl)ureido)pentyl)carbamoyl)-*L*-glutamic acid (RPS-039)

RPS-039 was synthesized from alkyne (**10**) by the same method and isolated as a white powder (34% yield). ^{1}H NMR (500 MHz, DMSO-d6) δ 8.30 (s, 1H), 8.09 (d, 1H, J = 9.7 Hz), 7.74 (s, 1H), 7.16 (d, 1H, J = 9.6 Hz), 6.95 (t, 1H, J = 5.4 Hz), 6.86 (m, 2H), 6.32 (m, 2H), 5.24 (s, 2H), 4.90 (t, 1H, J = 4.4 Hz), 4.78 (m, 2H), 4.72 (t, 1H, J = 4.4 Hz), 4.10 (m, 2H), 3.05 (m, 2H), 2.25 (m, 2H), 1.93 (m, 1H), 1.71 (m, 2H), 1.55 (m, 1H), 1.40 (m, 2H), 1.32 (m, 2H). ESI(+) = 582.4 $(M+H)^+$; ESI(-) = 580.3 $(M-H)^-$. Calculated mass: 581.22

(((*S*)-1-Carboxy-5-(3-(3-((1-(2-fluoroethyl)-1*H*-1,2,3-triazol-4-yl)methoxy)phenyl)ureido)pentyl)carbamoyl)-*L*-glutamic acid (RPS-043)

RPS-043 was synthesized from alkyne (**11**) by the same method and isolated as a white powder (60% yield). ^{1}H NMR (500 MHz, DMSO-d6) δ 8.40 (s, 1H), 8.25 (s, 1H), 7.18 (br s, 1H), 7.10 (t, 1H, J = 8.1 Hz), 6.89 (d, 1H, J = 8.1 Hz), 6.57 (d, 1H, J = 8.2 Hz), 6.31 (m, 2H), 6.13 (br s, 1H), 5.09 (s, 1H), 4.87 (t, 1H, J = 4.6 Hz), 4.76 (m, 2H), 4.69 (t, 1H, J = 4.6 Hz), 4.08 (m, 2H), 3.05 (m, 2H), 2.24 (m, 2H), 1.91 (m, 1H), 1.69 (m, 2H), 1.55 (m, 1H), 1.40 (m, 2H), 1.31 (m, 2H). ESI(+) = 582.4 $(M+H)^+$; ESI(-) = 580.2 $(M-H)^-$. Calculated mass: 581.22

(((*S*)-1-Carboxy-5-(3-(4-((1-(2-fluoroethyl)-1*H*-1,2,3-triazol-4-yl)methoxy)phenyl)ureido)pentyl)carbamoyl)-*L*-glutamic acid (RPS-038)

RPS-038 was synthesized from alkyne (**12**) by the same method and isolated as a white powder (77% yield). ^{1}H NMR (500 MHz, DMSO-d6) δ 8.27 (s, 1H), 8.25 (s, 1H), 7.30 (d, 2H, J = 9.0 Hz), 6.91 (d, 2H, J = 9.0 Hz), 6.34 (m, 2H), 6.10 (t, 1H, J = 5.3 Hz), 5.09 (s, 2H), 4.89 (t, 1H, J = 4.5 Hz), 4.78 (m, 2H), 4.71 (t, 1H, J = 4.5 Hz), 4.10 (m, 2H), 3.06 (m, 2H), 2.25 (m, 2H), 1.93 (m, 1H), 1.73 (m, 2H), 1.60 (m, 1H), 1.42 (m, 2H), 1.32 (m, 2H). ESI(+) = 582.3 $(M+H)^+$; ESI(-) = 580.3 $(M-H)^-$. Calculated mass: 581.22

Synthesis of 2-azidoethyltosylate

2-Azidoethanol (14)

Bromoethanol (250 mg, 2.0 mmol) was dissolved in H_2O (7 mL). A solution of sodium azide (195 mg, 3.0 mmol) in H_2O (3 mL) was added, and the reaction was stirred for 4 h at room temperature and then 16 h at 80°C. Then the reaction was cooled to room temperature and extracted with EtOAc. The organic layers were combined, dried over $MgSO_4$, filtered and concentrated under reduced pressure to give 2-azidoethanol (**14**) as a clear liquid (149 mg, 86% yield). ^{1}H NMR (500 MHz, $CDCl_3$) δ 3.88 (t, 2H, J = 5.1 Hz), 3.39 (t, 2H, J = 5.1 Hz), 3.14 (br s, 1H).

2-Azidoethyltosylate (15)

A solution of p-toluenesulfonyl chloride (394 mg, 2.07 mmol) in CH_2Cl_2 (5 mL) was added to a solution of 2-azidoethanol (149 mg, 1.72 mmol) in CH_2Cl_2 (10 mL). Triethylamine (0.48 mL, 3.44 mmol) was added, and the reaction was stirred for 5 h at room temperature under Ar. Then the reaction was washed successively with 1M HCl, H_2O and saturated NaCl solution. The organic layer was dried over $MgSO_4$, filtered and concentrated under reduced pressure to give a pale oil. The oil was purified by silica chromatography (33% EtOAc in hexanes) to give 2-azidoethyltosylate (**15**) as a colorless oil (204 mg, 49%

yield). ^{1}H NMR (500 MHz, $CDCl_3$) δ 7.74 (d, 2H, J = 8.2 Hz), 7.29 (d, 2H, J = 8.1 Hz), 4.08 (t, 2H, J = 5.1 Hz), 3.41 (t, 2H, J = 5.1 Hz), 2.39 (s, 3H).

Di-*tert*-butyl (1*H*-imidazole-1-carbonyl)-*L*-glutamate (17)

The hydrochloride salt of *L*-H-Glu(OtBu)-OtBu (1.25 g, 4.23 mmol) was dissolved in CH_2Cl_2 (30 mL) with a catalytic amount of *N*,*N*'-dimethylaminopyridine (50 mg), and the solution was cooled to 0°C and stirred under Ar. Triethylamine (4.5 mL) was added followed by 1,1'-carbonyldiimidazole (754 mg, 4.65 mmol), and the resulting mixture was stirred overnight with warming to room temperature. The reaction was then diluted with CH_2Cl_2 and washed successively with H_2O and saturated NaCl solution. The organic layer was dried over $MgSO_4$, filtered and concentrated under reduced pressure to give an oil that solidified upon standing. The crude product was purified by silica chromatography (100% EtOAc to 10% MeOH in EtOAc) to give the product, di-*tert*-butyl (1*H*-imidazole-1-carbonyl)-*L*-glutamate (**17**), as a transparent oil (1.00 g, 61% yield). ^{1}H NMR (500 MHz, $CDCl_3$) δ 8.18 (s, 1H), 7.48 (d, 1H, J = 6.2 Hz), 7.42 (s, 1H), 7.10 (s, 1H), 4.45 (m, 1H), 2.44 (m, 2H), 2.18 (m, 2H), 1.50 (s, 9H), 1.46 (s, 9H).

Tri-*tert*-butyl (9*S*,13*S*)-3,11-dioxo-1-phenyl-2-oxa-4,10,12-triazapentadecane-9,13,15-tricarboxylate (18)

A solution of di-*tert*-butyl (1*H*-imidazole-1-carbonyl)-*L*-glutamate (**17**) (572 mg, 1.48 mmol) in dichloroethane (6 mL) was cooled to 0°C and stirred under Ar. A solution of trimethylamine (0.42 mL, 3.0 mmol) in dichloroethane (1 mL) was added followed by a solution of methyl triflate (160 µL, 1.5 mmol) in dichloroethane. The reaction was stirred for 60 min, warming to room temperature. Then a solution of *L*-H-Lys(Cbz)-OtBu.HCl (552 mg, 1.48 mmol) in dichloroethane (10 mL) was added, and the reaction was stirred for 6 h at 50°C under Ar. The mixture was then cooled to room temperature and concentrated under reduced pressure to give an oil. The oil was purified by silica chromatography (20% EtOAC in hexanes to 50% EtOAc in hexanes) to give the product, tri-*tert*-butyl (9*S*,13*S*)-3,11-dioxo-1-phenyl-2-oxa-4,10,12-triazapentadecane-9,13,15-tricarboxylate (**18**), as a transparent oil (678 mg, 74% yield).

Di-*tert*-butyl (((*S*)-6-amino-1-(*tert*-butyloxy)-1-oxohexan-2-yl)carbamoyl)-*L*-glutamate (1)

Activated palladium on carbon (0.1 eq) was suspended in a solution of tri-*tert*-butyl (9*S*,13*S*)-3,11-dioxo-1-phenyl-2-oxa-4,10,12-triazapentadecane-9,13,15-tricarboxylate (**18**) (500 mg) in EtOH (15 mL). The suspension was stirred overnight at room temperature under H_2 atmosphere. The mixture was then filtered through celite, and the filtrate was concentrated under reduced pressure to give the product, di-

tert-butyl (((*S*)-6-amino-1-(*tert*-butyoxy)-1-oxohexan-2-yl)carbamoyl)-*L*-glutamate (**1**) as a viscous oil (360 mg, 92% yield).

Synthesis of DCFPyL

The synthesis of the cold ligand DCFPyL and the precursor trimethylammonium salt prosthetic group (**20**) were undertaken according to previously published procedures. [1,2]

_N_,_N_,_N_-Trimethyl-5-((2,3,5,6-tetrafluorophenoxy)-carbonyl)pyridine-2-aminium trifluoromethanesulfonate (20)

The title compound was isolated in three steps from 6-chloronicotinic acid as white crystals (137 mg, 19% yield). ^{1}H NMR (500 MHz, $CDCl_3$) δ 9.42 (d, 1H, J = 2.2 Hz), 8.94 (dd, 1H, J_1 = 8.7 Hz, J_2 = 2.2 Hz), 8.29 (d, 1H, J = 8.7 Hz), 7.57 (m, 1H), 3.76 (s, 9H). ESI(+) = 329.3 (M^+-OTf). Calculated mass: 329.09

6-Fluoronicotinic acid 2,3,5,6-tetrafluorophenyl ester (21)

The title compound was synthesized from trimethylammonium salt (**20**) as a white powder (2.5 mg, 14% yield). ^{1}H NMR (500 MHz, $CDCl_3$) δ 9.11 (d, 1H, J = 2.1 Hz), 8.59 (dt, 1H, J_1 = 8.2 Hz, J_2 = 2.4 Hz), 7.15 (dd, 1H, J_1 = 8.6 Hz, J_2 = 2.9 Hz), 7.10 (m, 1H).

2-(3-{1-Carboxy-5-[(6-fluoropyridine-3-carbonyl)-amino]-pentyl}-ureido)-pentanedioic acid (DCFPyL)

The title compound was synthesized from the activated ester (**21**) in two steps as a white powder (2.0 mg, 55% yield). ^{1}H NMR (500 MHz, DMSO-d6) δ 8.67 (m, 2H), 8.38 (m, 1H), 7.30 (dd, 1H, J_1 = 8.6 Hz, J_2 = 2.6 Hz), 6.33 (m, 2H), 4.08 (m, 2H), 3.26 (m, 2H), 2.25 (m, 2H), 1.93 (m, 1H), 1.72 (m, 2H), 1.58 (m, 3H), 1.36 (m, 2H). ESI(+) = 499.4 $(M+H)^+$. Calculated mass: 498.21

Radiosynthesis

[^{68}Ga]Ga-PSMA-HBED-CC

A 1.85 GBq ^{68}Ga/^{68}Ge Generator (ITG) was eluted with 4 mL 0.05M HCl, and $^{68}GaCl_3$ was obtained as a 185-222 MBq/mL solution. From this stock solution was taken 1 mL (containing approximately 185 MBq), which was combined with 5 µL of a 1 mg/mL solution of PSMA-HBED-CC (ABX) in H_2O at 95°C. The reaction was initiated by the addition of 20 µL of a 3N NaOAc solution, and heating to 95°C continued for 20 min on a Thermomixer. It was then passed through a pre-activated Sep-Pak Oasis™ cartridge (Waters), and the cartridge was washed with H_2O. [68Ga]Ga-PSMA-HBED-CC was eluted in a solution of 10% v/v EtOH in saline and diluted to a final concentration of approximately 100 MBq/mL. Decay-corrected radiochemical yield was greater than 95% and radiochemical purity was greater than 99%.

[^{18}F]DCFPyL *(n=1)*

10.73 GBq (290 mCi) [^{18}F]Fluoride in 2 mL $H_2{}^{18}O$ was dried azeotropically with MeCN at 100°C in the presence of 50 µL of a 100 µg/mL solution of KF in H_2O and 4 mg kryptofix-222. To the dried mixture was added 9 mg 6-trimethylammonium salt (**20**) in 1 mL MeCN, and the reaction was stirred at 40°C for 70 min. The reaction mixture was diluted with 10 mL and passed through a pre-activated Sep-Pak Silica cartridge (Waters). The eluate was evaporated to dryness at 60°C. To the dried mixture was added 1 mg di-*tert*-butyl (((*S*)-6-amino-1-(*tert*-butyoxy)-1-oxohexan-2-yl)carbamoyl)-*L*-glutamate (**1**) in 10 µL MeCN, 5 µL NEt_3 and 1 mL CH_2Cl_2. The reaction was stirred for 20 min at 40°C and then another 1 mg (**1**) in 10 µL MeCN and 5 µL NEt_3 were added. The reaction was stirred for a further 30 min before the solvent was evaporated and the crude product was dissolved in 100 µL TFA and stirred for 20 min at 40°C. The volatiles were evaporated under vacuum and the crude residue was dissolved in H_2O and purified by semi-prep HPLC (2 mL/min, 10 min gradient). The peak containing the product was collected, diluted with H_2O and trapped on a pre-activated Sep-Pak Oasis™ cartridge (Waters). The activity was eluted with 600 µL MeCN and concentrated at 100°C under vacuum. The crude residue was dissolved in 200 µL 0.9% NaCl solution. Total synthesis time was 230 minutes, and decay corrected radiochemical yield was 0.9%, radiochemical purity was greater than 96% and the specific activity was greater than 35 GBq/µmol.

References

1. Olberg DE, Arukwe JM, Grace D, Hjelstuen OK, Solbakken M, Kindberg GM, Cuthbertson A. One Step Radiosynthesis of 6-[^{18}F]Fluoronicotinic Acid 2,3,5,6-Tetrafluorophenyl Ester ([^{18}F]F-Py-TFP): A New Prosthetic Group for Efficient Labeling of Biomolecules with Fluorine-18. J Med Chem. 2010;53:1732-40.

2. Chen Y, Pallumbhatla M, Foss CA, Byun Y, Nimmagadda S, Senthamizhchelvan S, et al. 2-(3-{1-Carboxy-5-[(6-[^{18}F]Fluoro-Pyridine-3-Carbonyl)-Amino]-Pentyl}-Ureido)-Pentanedioic Acid, [^{18}F]DCFPyL, a PSMA-Based PET Imaging Agent for Prostate Cancer. Clin Cancer Res. 2011;17:7645-53.
